# Thermal Plasticity is Regulated by a Key MicroRNA During Range Expansion of an Invasive Fruit Fly

**DOI:** 10.1002/advs.202507662

**Published:** 2026-02-24

**Authors:** Yan Zhao, Juntao Hu, Juan Du, Shaokun Guo, Youming Hou, Zhihong Li

**Affiliations:** ^1^ State Key Laboratory of Agricultural and Forestry Biosecurity MARA Key Lab of Surveillance and Management for Plant Quarantine Pests College of Plant Protection China Agricultural University Beijing China; ^2^ State Key Laboratory of Agricultural and Forestry Biosecurity Fujian Agriculture and Forestry University Fuzhou China; ^3^ Ministry of Education Key Laboratory for Biodiversity Science and Ecological Engineering Institute of Biodiversity Science Center for Evolutionary Biology School of Life Sciences Fudan University Shanghai China

**Keywords:** genetic assimilation, invasion mechanism, invasive fruit fly, microRNA regulation, range expansion, thermal plasticity

## Abstract

Understanding the mechanisms that enable invasive species to expand into novel thermal environments is key to predict their future distribution range under climate change. Plasticity is a key driver behind range expansion during invasion, yet the post‐transcriptional regulatory mechanism underlying plasticity during range expansion remains less explored. Here, we performed an integr analysis of phenotypic, transcriptomic, and microRNA (miRNA) changes in a range‐expanding invasive insect, *Bactrocera dorsalis*, under heat and cold acclimation. We found that populations at the invasive front exhibited reduced plasticity in fitness‐related traits that were corroborated by genetic assimilation of frontloaded genes. Weighted Gene Co‐expression Network Analysis uncovered important modules associated with acute cold tolerance of *B. dorsalis* and indicated *thw* gene as a critical network component. Furthermore, *thw* was found to be regulated by a key miRNA, miR‐276b, with its function verified by our dual‐luciferase reporter assay and RNAi‐mediated knockdown experiment. Our findings suggested that miRNA‐mediated regulation of plasticity might be key to allow invasive species to expand into novel thermal environments.

## Graphical abstract

Populations at the invasion front of *Bactrocera dorsalis* adapt through reduced phenotypic and gene expression plasticity, a phenomenon likely to be driven by genetic assimilation. We identify a key miRNA‐mediated regulatory axis, in which miR‐276b post‐transcriptionally represses *thw*, a conserved chitin‐binding gene critical to the cold‐tolerance network, thereby revealing a molecular mechanism that facilitates thermal adaptation during range expansion.

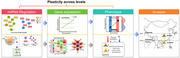

## 1 Introduction

The ongoing climate change due to increasing anthropogenic activities has caused shifts in distribution ranges of many invasive species, with temperature shifts serving as a critical driver of these changes [1, 2, 3], particularly for ectothermic invaders whose physiology and life‐history traits are closely linked to thermal conditions [4, 5, 6]. As global temperatures rise, invasive species are increasingly expanding their ranges toward higher latitudes and altitudes, often accompanied by significant phenotypic changes [7, 8]. Notable examples include that invasive *Asteraceae* species exhibit reduced body size and fewer inflorescences at higher altitudes [9, 10], cane toads *Rhinella marina* in Australia display gradient‐dependent dispersal behaviors [11, 12], and *Lonomia obliqua* populations in the United States show distinct thermal adaptations, such as larger body sizes in warmer regions and enhanced cold tolerance in cooler areas [13, 14]. Understanding the mechanisms of thermal stress responses, particularly those enhancing environmental fluctuation tolerance, is critical for elucidating how invasive species adapt to thermal variability, thereby driving distribution shifts and facilitating their successful colonization in new thermal habitats [15, 16, 17].

Key mechanisms that enable this phenotypic variation in response to environmental changes include evolutionary change or existing phenotypic plasticity [18, 19]. However, the rapid adaptation observed in many species, particularly in populations with limited genetic diversity, cannot be fully explained by genetic mutations alone [20, 21, 22]. Instead, plasticity provides an important mechanism for populations to enhance tolerance, and cope with increasingly variable and extreme temperatures [23]. Plasticity is defined as the ability of a genotype to produce different phenotypes in response to environmental changes [24]. Such plasticity can be achieved through acclimation, whereby prior thermal exposure shifts thermal tolerance, allowing organisms to perform better or recover effectively at extreme temperatures [25, 26]. Importantly, plasticity can either constrain evolution by buffering phenotypic selection or instead facilitate adaptive evolution through genetic accommodation [27, 28, 29]. Furthermore, plasticity itself may evolve, with examples such as “frontloading”, where more tolerant populations exhibit higher baseline expression and reduced plasticity under new conditions [30]. If advantageous, this could lead to genetic assimilation, assuming the ancestral states exhibited higher plasticity [31, 32], as observed in long‐term adaptation to high CO_2_ in the cyanobacterium *Trichodesmium* [33], maize domestication [34], and developmental plasticity in Solanum [35]. Despite its importance, the interplay between plasticity and evolution remains understudied in natural populations [19, 29].

Addressing this gap, transcriptomic studies have provided valuable insights into the mechanisms underlying plasticity and adaptation, particularly through gene expression changes in response to environmental stressors [36, 37, 38]. For example, southern populations of the killifish *Fundulus heteroclitus* exhibit greater thermal tolerance and stronger plasticity of heat shock genes compared to northern populations [39], while trinidadian guppies *Poecilia reticulata* from high or low predation environments show evolving expression plasticity across populations [40]. Similarly, *Ischnura elegans* damselflies show genetic compensation under warming conditions [41]. These examples highlight the functional role of gene expression plasticity in adapting to changing environments [42, 43]. However, interpreting transcriptomic data to link gene expression to stress responses remains challenging due to its dynamic nature, and approaches such as reaction norm frameworks [44, 45] and co‐expression network analyses (e.g., WGCNA) [46] offer promising avenues to elucidate these connections. Recent studies have also highlighted the role of post‐transcriptional regulation, particularly microRNAs (miRNAs), in regulating gene expression in response to environmental stresses such as extreme temperature [47, 48, 49]. miRNA, small RNAs of approximately 22 nucleotides (nt), repress messenger RNA (mRNA) targets [50], and can promote plastic responses by regulating the expression of target genes [51, 52]. Despite their potential importance, the extent to which miRNAs regulate plasticity and its evolution in natural populations, particularly in invasive species, remains poorly understood. Investigating miRNA‐mediated regulatory mechanisms could provide critical insights into how invasive species adapt to novel temperature environments, offering a promising direction for future research [53, 54].

This is particularly relevant for recently introduced populations, which frequently have reduced genetic diversity due to bottlenecks and founder effects [55, 56], making phenotypic plasticity a key driver of range expansion during invasions [57, 58]. Invasive species, which frequently encounter diverse environmental stresses, generally exhibit greater phenotypic plasticity in growth, morphology, and physiology compared to non‐invasive species [29, 59, 60]. Thus, they provide great opportunities to study the impact of environmental changes on gene expression plasticity and regulatory mechanisms. We focused on the Oriental fruit fly *Bactrocera dorsalis* (Hendel) (Diptera: Tephritidae), a notorious invasive species with a wide latitudinal and temperature range across China, currently expanding to higher latitudes [61, 62]. Previous studies have demonstrated distinct latitude‐associated differences in thermal tolerance between cold‐adapted northern marginal populations and warm‐adapted southern populations of *B. dorsalis* in China [63]. Despite these differences, genetic analyses, including microsatellites and genome‐wide SNPs, revealed minimal genetic variation among Chinese populations [64, 65, 66], suggesting that phenotypic divergence is unlikely driven by genetic selection, and is more likely due to adaptive plasticity. While phenotypic changes in *B. dorsalis* in response to new thermal environments have been documented, the interplay between plasticity and evolutionary mechanisms underlying these changes, particularly the regulatory role of microRNAs (miRNAs), remains poorly understood. To address these gaps, we compared southern ancestral and northern derived populations of *B. dorsalis* acclimated under cold and warm treatments. We assessed thermal plasticity using multiple phenotypic indicators, and analyzed gene expression miRNAs profiles in head and muscle tissues to identify plasticity patterns and regulatory networks. We hypothesized that northern populations at the invasion front would exhibit decreased phenotypic plasticity, corresponding to changes in gene expression plasticity. If miRNAs play a role in the evolution of gene expression plasticity, we expected to identify relevant regulatory networks through combined RNA‐seq and miRNA‐seq analyses. Our findings will provide insights into the role of gene expression plasticity in shaping thermal adaptation and the potential contribution of miRNAs to phenotypic divergence during biological invasions.

## 2 Results

### 2.1 Phenotypic Divergence among Populations in Response to Thermal Acclimation

#### 2.1.1 Critical Thermal Limit

**FIGURE 1:**
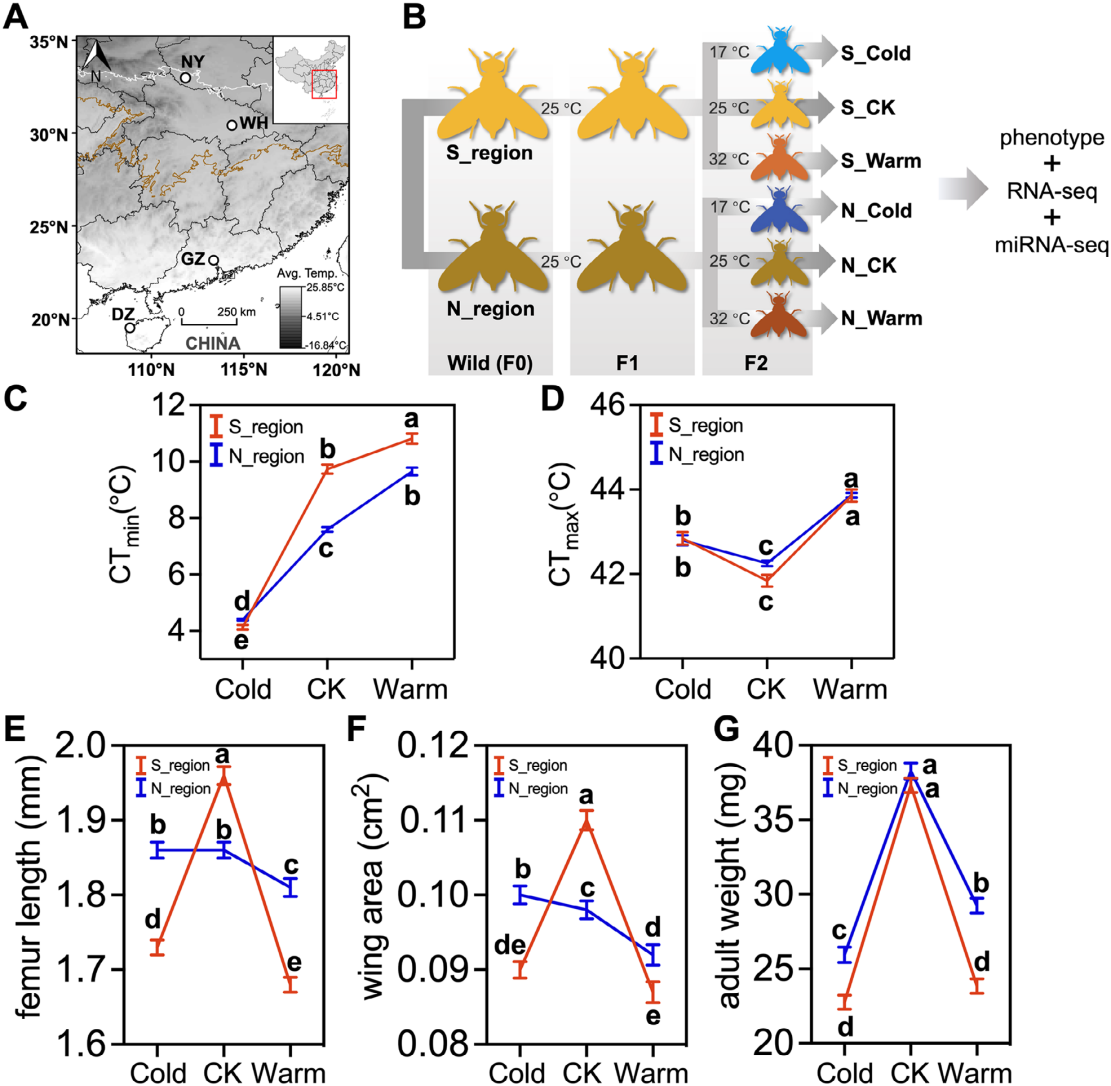
(A) Geographic sampling locations of *Bactrocera dorsalis* used in this study. DZ, Danzhou, Hainan Province; GZ, Guangzhou, Guangdong Province; WH, Wuhan, Hubei Province; NY, Nanyang, Henan Province. Color tone indicates the local average annual temperature. The white and brown lines showed the critical overwintering area and the general overwintering area of *B. dorsalis* in China, respectively. (B) Experimental design outlining regional sampling and thermal acclimation procedures. Samples were initially acclimatized under optimum conditions (25°C) in a laboratory common garden through one generation (F1). Then, samples were exposed to warm (32°C), cold (17°C), or control (25°C) conditions, respectively, and reared at these temperatures until reaching sexual maturity (F2). (C) CT_min_ (n ≈ 80, *F*_(5,470)_ = 509.1, *p* < 0.0001, one‐way ANOVA), (D) CT_max_ (n ≈ 75, *F*_(5,444)_ = 53.59, *p* < 0.0001, one‐way ANOVA), (E) femur length (n ≈ 105, *F*_(5,624)_ = 73.65, *p* < 0.0001, one‐way ANOVA), (F) wing area (n ≈ 60, *F*_(5, 338)_ = 43.17, *p* < 0.0001, one‐way ANOVA), and (G) adult weight (n ≈ 300, *F*_(5,1792)_ = 156.7, *p* < 0.0001, one‐way ANOVA) of *B. dorsalis* in southern and northern regions after one‐generation acclimation at cold, warm, or control temperatures. Detailed mean values and standard errors (mean ± SEM) were provided in Table S1. To assess the main and interactive effects of region and thermal acclimation, a two‐way ANOVA was performed on the pooled data from both regions (full statistical results in Table S2).

Cold tolerance significantly differed between the two regions (Figure 1C; Table S1), with the northern population showing lower the critical thermal minimum (CT_min_) compared to the southern population. The critical thermal maximum (CT_max_), however, did not differ significantly between the regions (Figure 1D; Table S1). Region, thermal acclimation, and their interaction all significantly associated with cold tolerance (Table S2). Cold acclimation significantly improved CT_min_ in both populations. The southern population reflected higher plasticity, which led to greater cold tolerance than the northern population under cold acclimation Warm acclimation, conversely, significantly compromised CT_min_ in both populations. Thermal acclimation also significantly affected CT_max_, but the magnitude of change was similar between the two regions. Individuals subjected to thermal acclimation exhibited significantly elevated heat tolerance, with the highest CT_max_ recorded after warm acclimation, followed by cold acclimation. Overall, the northern population had stronger basal cold tolerance, whereas the southern population exhibited greater plasticity after thermal acclimation.

#### 2.1.2 Adult Size

Both body size traits (femur length and wing area) were significantly larger in the southern population than in the northern population (Figure 1E,F; Table S1). Cold acclimation significantly reduced both femur length and wing area in the southern population. In the northern population, femur length showed no significant change, while wing area increased significantly under cold acclimation. Warm acclimation significantly decreased the two traits in both populations, with greater reductions in the southern population, resulting in a significantly smaller body size than the northern population. Furthermore, a significant region × acclimation interaction effect was observed for both traits (Table S2). In summary, the southern population exhibited a larger body size, and greater plasticity under thermal acclimation.

#### 2.1.3 Body Mass

There was no significant difference in adult weight between the two populations (Figure 1G; Table S1). Both cold and warm acclimation significantly reduced the adult weight in both populations, with the southern population recording a lower adult weight. This suggested that the southern population exhibited higher plasticity in adult weight in response to thermal acclimation. Additionally, a significant region × acclimation interaction effect on adult weight was observed (Table S2).

### 2.2 Global and Differential Gene Expression Pattern

**FIGURE 2:**
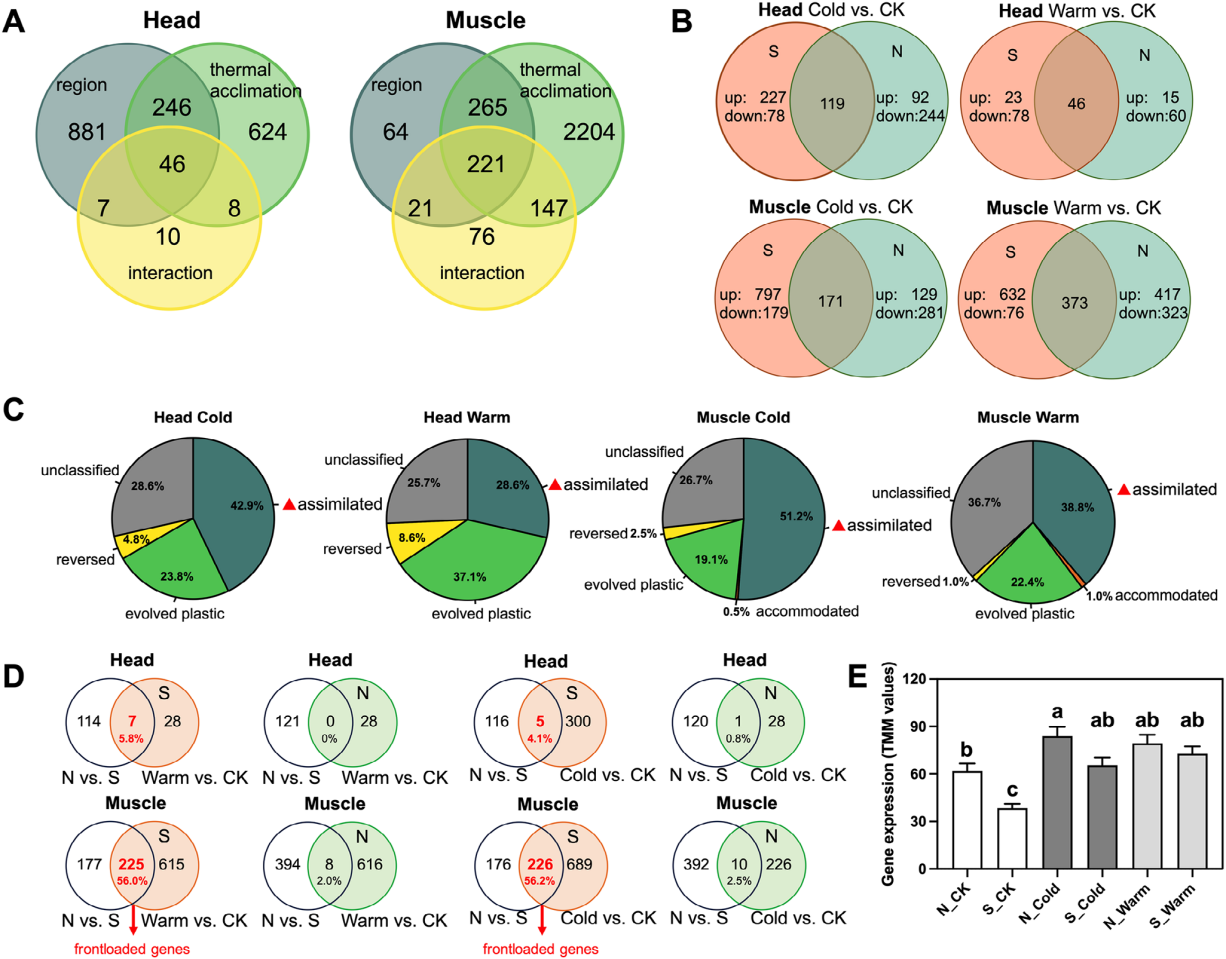
(A) Overview of differential expression analysis results. Numerous transcripts in both head (left) and muscle (right) tissues exhibited differential expression due to region (dark green), acclimation temperature (light green), and their interaction (yellow). (B) Venn diagrams of genes differentially expressed between exposure temperatures within a given region. Differentially expressed genes were identified using a *p*_adj_ cut‐off of 0.05. (C) Evolution of transcript expression plasticity based on Renn and Schumer (2013) (Figure S2). All categories underwent *Z*‐test analysis (*p* < 0.05). (D) Venn diagrams showing the number of upregulated genes in the northern population (vs. southern population) under the control condition and upregulated genes detected during analysis based on within‐region thermal response. (E) Expression of genes that were upregulated in the northern population (vs. southern population) under control conditions in muscle (n = 402, *F*_(5, 2406)_ = 11.36, *p* < 0.0001, one‐way ANOVA). Y‐axis: normalized gene expression (TMM). X‐axis: six cDNA libraries of muscle. Region: northern‐edge (N), southern‐edge (S); cold acclimation (Cold), warm acclimation (Warm), control condition (CK).

An average of 24.5 million read pairs were generated for the 70 transcriptome libraries. After preprocessing, an average of 10.4 million high‐quality reads per sample were uniquely mapped to the reference genome and retained for downstream analysis (Tables S3 and S4). After filtering out weakly expressed genes, 9645 transcripts remained for differential expression analysis. Initial principal component analysis (PCA) clustering of the overall transcriptome profiles revealed that samples clustered by tissue type (Figure S5A), with muscle exhibiting greater gene expression variance than head tissue. Differential expression analysis further revealed a pronounced tissue‐specific response (Figure 2A; Dataset S1). The total number of differentially expressed genes (DEGs) in head was significantly lower than in muscle (1,822 vs. 2,998; χ^2^(1) = 382.63, *p* < 0.0001, chi‐squared test). In head, most DEGs were associated with regional genetic differences, while a smaller set responded to thermal acclimation, and only a minimal number showed a region‐by‐acclimation interaction. In contrast, muscle displayed a strong, acclimation‐driven transcriptional response, alongside more modest regional differences and a notable set of interaction genes. This weaker response of the thermal transcriptional response in head is likely due to the fact that neural and sensory functions may be buffered against direct temperature fluctuation or regulated through alternative, non‐transcriptional mechanisms.

Venn diagrams visualized the overlap of DEGs between geographic regions under each acclimation condition (Figure 2B). In head, a moderate proportion of cold‐ (15.7%) and warm‐responsive (20.7%) DEGs were shared between regions. A comparable trend appeared in muscle, with a higher proportion of shared genes under warm (20.5%) than under cold acclimation (11.0%). In addition, the transcriptomic response was consistent with the greater phenotypic plasticity observed in the southern population (Figure 1C–G). Specifically, the southern population exhibited a markedly stronger transcriptional response to cold acclimation (976 DEGs) than the northern population (410 DEGs), whereas both populations showed a comparable scale of unique gene expression changes in response to warm acclimation (708 vs. 740).

### 2.3 Evolution of Expression Plasticity

We identified more genes showing evolution of expression plasticity in muscle (465 genes) than in head (71 genes), accounting for 15.51% and 3.9% of the tissue‐specific DEGs, respectively (Figure 2A). Across both tissues and acclimation conditions, the majority of transcripts exhibiting evolved plasticity followed a genetic assimilated pattern (head‐cold: 42.9%, *Z* = 2.673, *p* < 0.0001; muscle‐cold: 51.2%, *Z* = 16.220, *p* < 0.0001; muscle‐warm: 38.8%, *Z* = 4.647, *p* < 0.0001, *Z*‐test), with the exception being the warm‐acclimated genes in head (28.6%, *Z* = 0.488, *p* = 0.625, *Z*‐test; Figure 2C). Functional enrichment analysis revealed distinct tissue profiles. In head, no specific Gene Ontology (GO) categories were significantly enriched among assimilated genes. Notably, one gene related to chitin binding, peritrophin‐44, exhibited assimilation under both warm and cold acclimation. In contrast, assimilated genes in muscle were functionally coordinated, showing significant enrichment for processes such as active transmembrane transporter activity (GO:0022804) in response to cold, and positive regulation of kinase activity (GO:0033674) in response to warm acclimation (Dataset S2).

Genes with higher baseline expression in the northern population under control conditions were extensively upregulated in the southern population during thermal acclimation, but almost no further upregulation occurred in the northern population. Specifically, 56.0% (warm) and 56.2% (cold) of these genes were upregulated in the southern population, compared to only 2.0% and 2.5% in the northern population, respectively (Figure 2D). Expression profiles confirmed that these genes, initially expressed at lower levels in the southern population, were induced during acclimation to reach expression levels comparable to the northern baseline (Figure 2E). This pattern suggests that the northern population, inhabiting a colder and more thermally variable environment, tends to constitutively express or “frontload” a set of genes that are only induced in the southern population upon thermal acclimation. Consistent with our phenotypic results showing greater baseline tolerance in the northern population and greater acclimation plasticity in the southern population (Figure 1C–G), these frontloaded genes appear to be pre‐activated in the tolerant northern population, thereby providing a rapid‐response capacity, whereas their inducible expression in the sensitive southern population contributes to its plastic acclimation response.

### 2.4 Gene Coexpression Modules Associated with Thermal Tolerance

**FIGURE 3:**
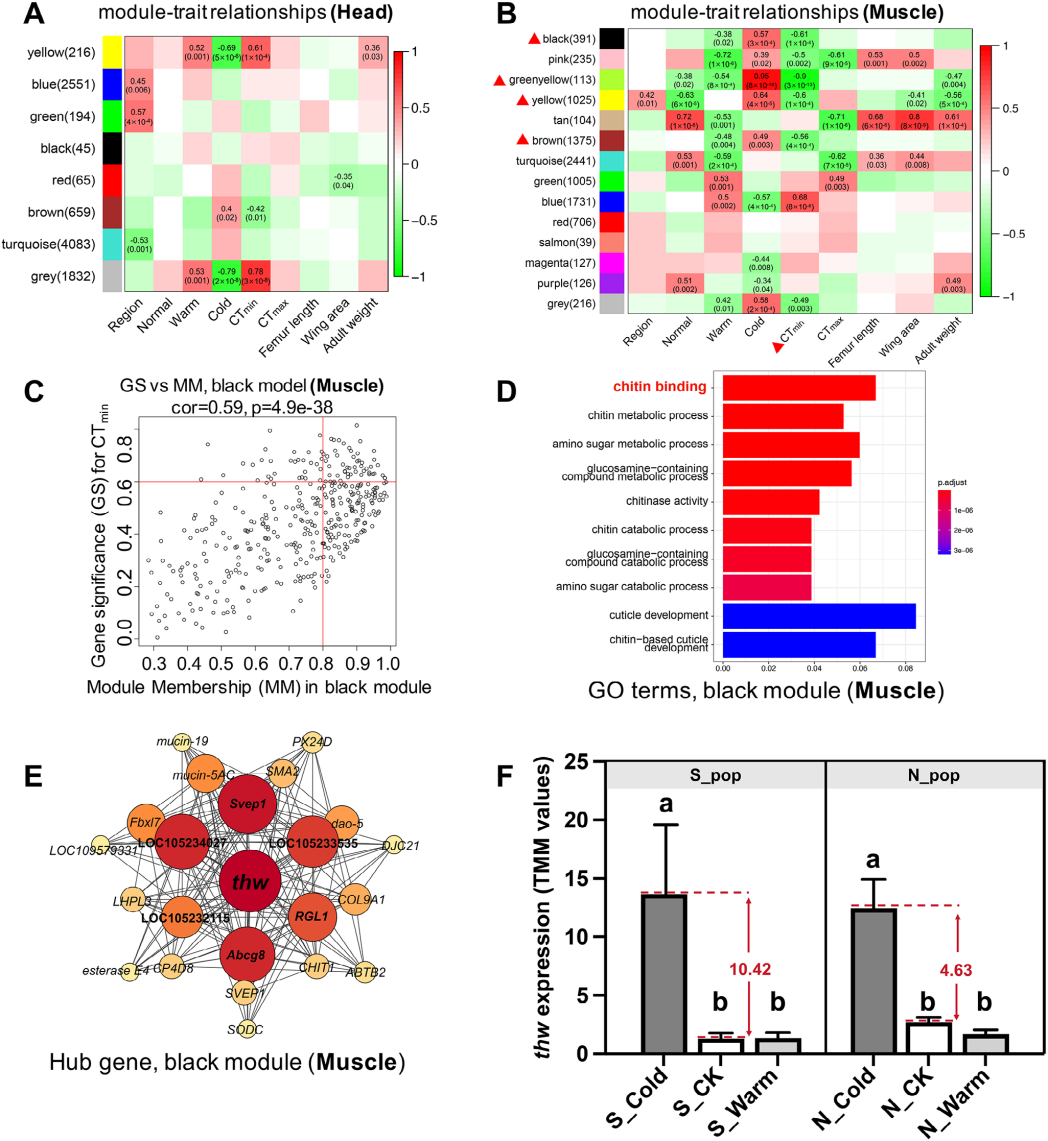
Association analysis between eigengene expression and qualitative traits. Clustering of module eigengenes and heat maps of module‐trait correlations for (A) head and (B) muscle tissues. Numbers in parentheses indicate gene count in each module. Significant relationships between module eigengenes (rows) and treatments (columns) are illustrated by the colour scale (red‐green), representing the strength of the correlation (1 to ‐1). Each correlation is represented by the correlation coefficient (r) and its *p*‐value (in parentheses). (C) Scatterplot of gene significance (GS) for CT_min_ vs. module membership (MM) in the black module. GS and MM exhibit a significant correlation, suggesting hub genes in the black module are closely linked to CT_min_. (D) Top 10 over‐represented GO terms in the black module. Corrected *p*‐values are indicated by color (warmer colors signify lower *p*‐values). The vertical axis represents GO terms, and the horizontal axis shows gene ratios. (E) The coexpression network of hub genes with MM > 0.8 and GS > 0.8 in the black module (Dataset S5). Nodes represent genes, and edges (lines) represent gene interactions. The importance of hub genes is ranked by connection, indicated by color depth and circle size, with topological overlap of the connections is above 0.08. (F) Expression patterns of the *thw* gene in response to thermal acclimation. Bar diagram showed normalized gene expression (TMM) of *thw* across the southern population (n = 6, *F*_(2,15)_ = 5.94, *p* = 0.013, one‐way ANOVA) and northern population (n = 6, *F*_(2,15)_ = 38.68, *p* < 0.0001, one‐way ANOVA).

Weighted Gene Co‐expression Network Analysis (WGCNA) partitioned all transcripts into eight (head) and 14 (muscle) modules, each designated by a unique eigengene (Figure 3A,B). We identified one module in head (yellow), and four modules in muscle (black, greenyellow, yellow, and brown) that showed significant negative correlation with CT_min_ (*p* < 0.05, correlation coefficient < −0.5). No modules were significantly positively correlated with CT_max_ in either tissue. Although the head yellow module contained genes with diverse functions, including basic cellular regulation and neuroendocrine signaling (Dataset S3), it showed no significant GO category enrichment. Therefore, we focused on muscle for further analysis. The four cold‑tolerance‑associated modules in muscle (black, green‐yellow, yellow, and brown) exhibited robust correlations with CT_min_ (R^2^ ranging from −0.56 to −0.90, all *p* < 0.001). High concordance between gene significance (GS) and module membership (MM) was observed for green‐yellow (cor = 0.89), and black (cor = 0.59) modules (Figure 3C; Figure S7), confirming their strong relevance to the trait (cor(GS vs. MM) > 0.5, *p* < 0.05).

GO enrichment analysis (Dataset S4) revealed that the green‐yellow module was enriched with pyrimidine‐containing compound metabolic process (GO:0072527). In contrast, the black module was enriched in processes directly related to thermal adaptation (Figure 3D), including chitin binding (GO:0008061), chitin‐based cuticle development (GO:0040003), and related metabolic processes (e.g., chitin metabolic process, GO:0006032), consistent with known mechanisms of insect thermal tolerance [67, 68, 69]. Based on its functional signature, we identified the black module as a putative transcriptional signature induced of thermal stress in *B. dorsalis*. Network visualization in Cytoscape revealed 23 hub genes (MM > 0.8 and GS > 0.8; Figure 3C,E) likely to constitute a core regulatory network for thermal adaptation. Among these, *thw*, a chitin‑binding gene, emerged as the most central node (Figure 3E). Expression analysis further revealed that *thw* was significantly induced by cold acclimation in both populations Interestingly, the cold‐induced plastic response in the *thw* gene was greater in the southern population than the northern population (fold‐change, southern vs. northern: 10.42 vs. 4.63; Figure 3F).

### 2.5 Identification of miRNAs and Differential Expression of miRNAs

An average of 27.5 million reads per sample passed quality and length filters, with 66.0% mapping to the reference genome (Tables S3 and S4). The small RNA length distribution peaked at 22nt, the typical length of mature miRNAs [70], and a high frequency of U (54.48%) was observed at the 5’ end (Figure S8A,B), consistent with previous findings [71]. We identified 145 high‐confidence miRNAs in *B. dorsalis* using miRDeep2 and stringent filtering (Dataset S6), including 93 novel miRNAs, which were comparable to those reported by Peng et al. [72] Sixteen of these were conserved in *Drosophila melanogaster*, with miR‐278‐3p, miR‐987‐5p, and miR‐317‐3p showing high sequence conservation (Figure S8C). Predicted miRNAs precursors displayed canonical stem‐loop structure and typical strand bias (Figure S8D,E). The miRNAs were distributed roughly equal numbers in intergenic (43.3%) and genic regions (56.7%), with most genic miRNAs located in introns (84.2%; Figure S8F), aligning with previously observed patterns [51].

**FIGURE 4:**
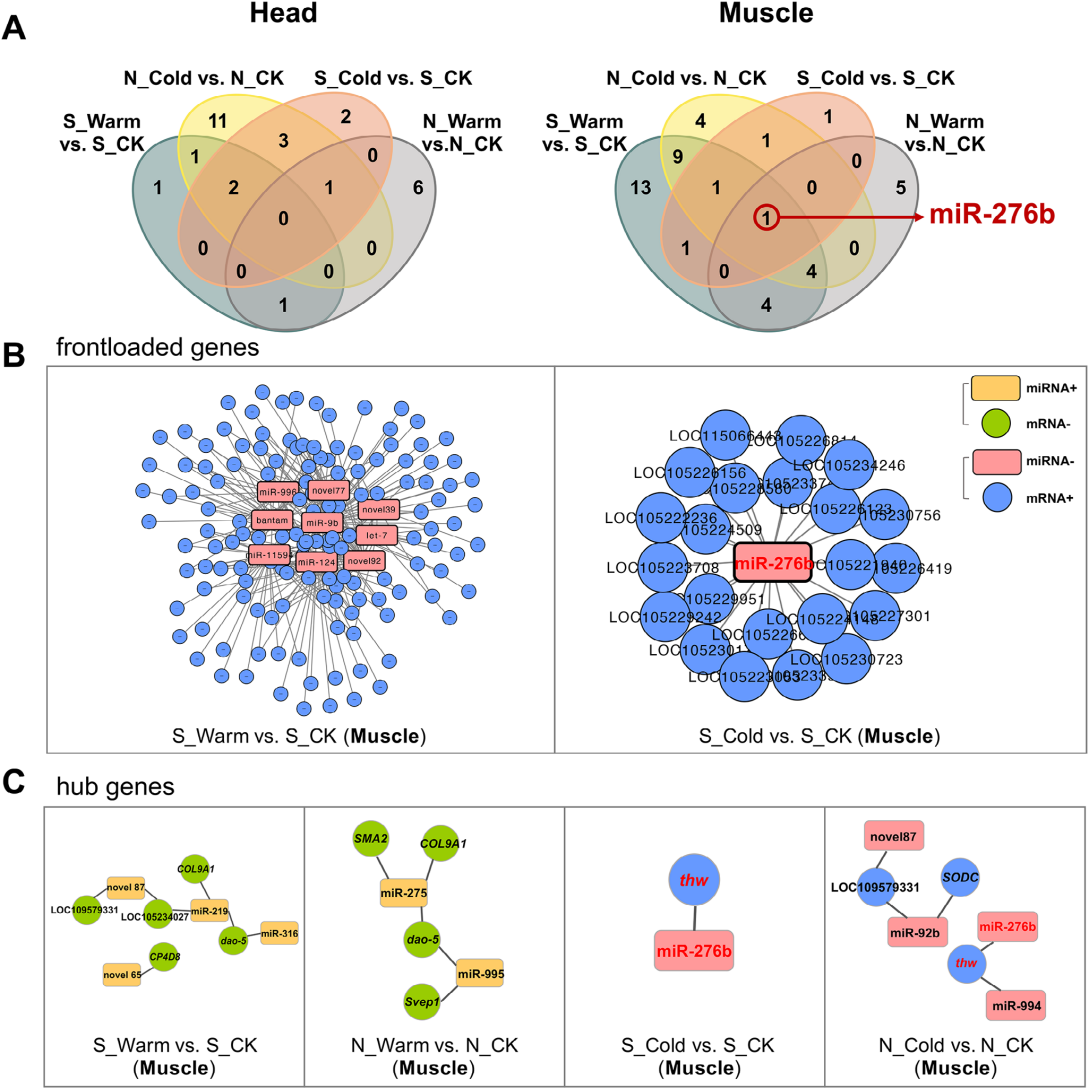
(A) Venn diagrams of genes differentially expressed in response to thermal acclimation in northern and southern regions in head and muscle. Differentially expressed miRNAs were identified using a *p*_adj_ cut‐off of 0.05. (B) Predicted interaction network between miRNAs and frontloaded genes shown in Figure 2D. (C) Predicted interaction network between miRNAs and hub genes in the black module shown in Figure 3E. Specific gene information was shown in Dataset S9.

A total of 81 mature miRNAs passed expression thresholds and were examined for differential expression. PCA clearly separated head and muscle samples (PC1 explained 93.7% variance; Figure S5B). We detected more differentially expressed miRNAs in muscle (44 total: 32 upregulated, 27 downregulated) than in head (34 total: 16 upregulated, 22 downregulated; Dataset S7). Notably, miR‐276b was the only miRNA that responded significantly to warm and cold acclimation in both regions within muscle, suggesting a key regulator in thermal acclimation (Figure 4A).

### 2.6 miRNA‐mRNA Networks Regulating Genes Involved in Expression Plasticity and Thermal Acclimation

To explore the regulatory basis of the phenotypic and transcriptomic differences, we first evaluated population genetic structure using SNPs extracted from RNA‐seq data. Genetic differentiation between southern and northern populations was low (*F*_ST_ = 0.0196), with no clear geographic clustering in PCA or ADMIXTURE analysis (Figure S9), driver of phenotypic and transcriptomic differences other than genetic variation between northern and southern populations. We therefore focused on miRNA‑mediated post‑transcriptional regulation. Each putative thermal‐responsive miRNAs had 478 putative targets on average, totaling 28 998 and 31 413 targets, corresponding to 8877 and 9293 unique genes in head and muscle, respectively. After filtering for negatively correlated expression (r < −0.4, Dataset S8), these miRNAs were linked to 16.16% (head) and 22.01% (muscle) of genes affected by thermal acclimation and region (Figure S10), indicating their extensive involvement in the transcriptional response to temperature.

In the southern population, downregulated miRNAs regulated the upregulation of 62.22% (warm) and 9.73% (cold) of frontloaded genes (Figure 4B). Warm acclimation involved nine downregulated miRNAs targeting 140 genes, while under cold acclimation, only miR‐276b targeted 22 genes, suggesting that miRNAs downregulation may release transcripts from repression to promote rapid translation. Analysis of miRNAs targeting genes with significant expression plasticity further revealed that in muscle, four upregulated miRNAs potentially regulated five assimilated genes, while five downregulated miRNAs targeted three assimilated genes (Figure S11; Dataset S9). Within the thermal‐response network, 43.5% of the 23 previously identified hub genes were predicted targets of thermally responsive miRNAs, with targeting patterns varying between acclimation conditions and populations (Figure 4C; Dataset S9). Notably, miR‐276b was predicted to regulate *thw* under cold acclimation in both populations, highlighting its potential as a key post‑transcriptional regulator in thermal acclimation of *B. dorsalis*.

### 2.7 miR‐276b Regulates Cold Tolerance in *Bactrocera Dorsalis* by Negatively Targeting *thw*

We first analyzed SNP patterns in the *thw* genomic region using RNA‑seq data. No fixed differences were found between populations (Figure S12), suggesting that sequence variation in this region is unlikely to explain the differential cold‑induction of *thw*. Evolutionary analysis revealed that the THW protein contains a highly conserved CBM_14 domain (Figure S13A), which serves as the core chitin‐binding module of the Peritrophin‐A domain. Multiple sequence alignment demonstrated extensive conservation of this domain across 16 insect species, particularly the six cysteine residues forming three structurally critical disulfide bridges (Figure S13A). Phylogenetic analysis showed THW orthologs from Diptera formed a distinct cluster with high bootstrap support (Figure S13B), suggesting evolutionary conservation within this order. Structural comparison with the Peritrophin‐A domain from *Popillia japonica* confirmed the preservation of essential chitin‐binding residues (F/Y) in the CBM_14 domain (Figure S13C). These residues mediate interactions with N‐acetylglucosamine (GlcNAc) units through hydrophobic interactions, hydrogen bonding, and stacking, likely underlying THW's functional role in thermal tolerance.

**FIGURE 5:**
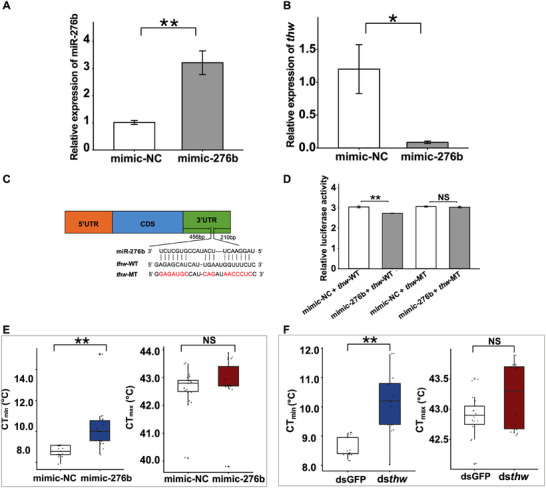
The target genes of miR‐276b and its regulatory role in thermal tolerance in *Bactrocera dorsalis*. Relative expression of (A) miR‐276b (n = 6, *p* = 0.007, Independent sample *t*‐test) and (B) potential target genes after mimic‐276b injection, with 18s as the control gene (n = 12, *p* < 0.0001, Independent sample *t*‐test). (C) Binding region information for *thw*. (D) Dual luciferase reporter assays in wild (n = 5, *p* = 0.0019, Independent sample *t*‐test) and mutant types (n = 5, *p* = 0.543, Independent sample *t*‐test) of *thw* using HEK293T cells co‐transfected with miRNA mimic and pmirGLO/recombinant pmirGLO vectors containing the predicted or mutant binding sites. (E) CT_min_ (n = 30, *p* < 0.0001, Independent sample *t*‐test) and CT_max_ (n = 30, *p* = 0.74, Independent sample *t*‐test) of *B. dorsalis* after mimic‐276b injection. (F) CT_min_ (n = 30, *p* < 0.0001, Independent sample *t*‐test) and CT_max_ (n = 30, *p* = 0.057, Independent sample *t*‐test) of *B. dorsalis* after ds*thw* and dsGFP injection. Data are presented as mean values and standard errors (mean ± SEM). Asterisks “^*^” and “^**^” above the bars represent significant differences at *p* < 0.05 and *p* < 0.01, respectively, and “NS” represents no significant differences.

We then investigated miR‐276b‐mediated regulation of *thw*. Overexpression of miR‐276b mimics resulted in a 2.12‐fold increase in miR‐276b levels and a 92.93% reduction in *thw* expression (Figure 5A,B), demonstrating potent post‐transcriptional repression. Both bioinformatic prediction and experimental validation confirmed that miR‐276b targeted the 3’ untranslated region (UTR) of *thw* (Figure 5C; Figure S15). Dual‐luciferase reporter assays in HEK293T cells showed a 9.8% reduction in luciferase activity that was abolished by seed sequence mutations (Figure 5D), establishing direct miR‐276b‐mediated repression of *thw*. Functional characterization revealed that injection of mimic‐276b significantly increased adult CT_min_ by 16% while CT_max_ remained unchanged (Figure 5E). Similarly, RNAi‐mediated knockdown of *thw* (40.97% reduction, Figure S16) increased CT_min_ by 18% (Figure 5F), with no effect on CT_max_ (Figure 5F), confirming that cold tolerance was regulated by *thw* expression levels, under miR‐276b regulation.

**FIGURE 6:**
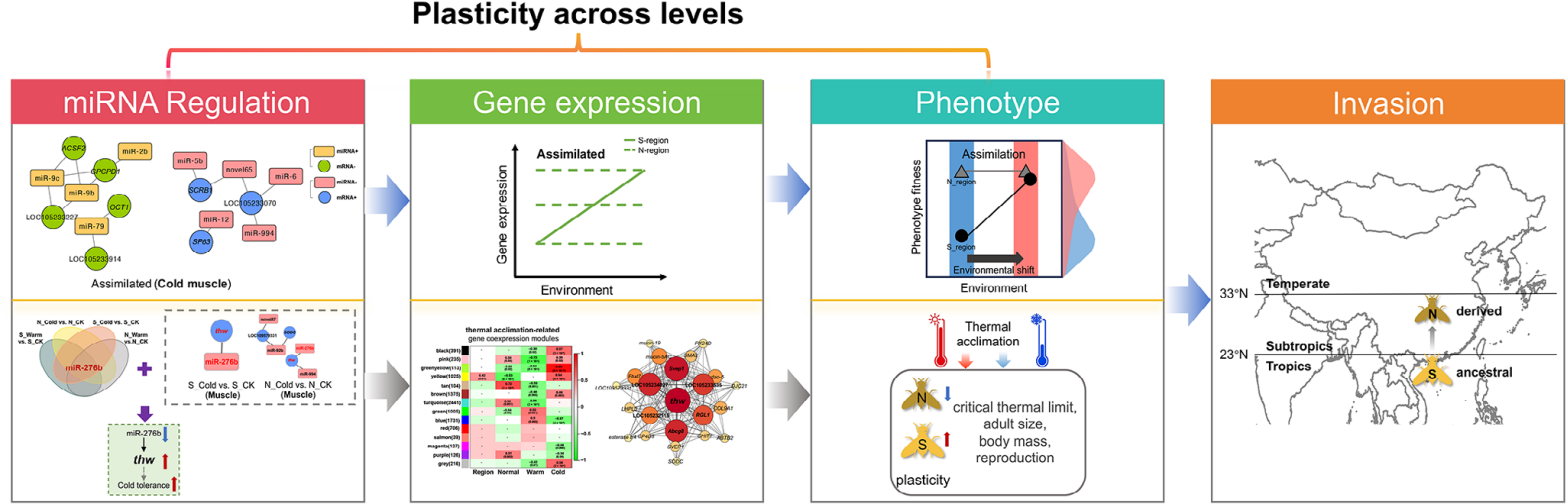
Molecular model of miRNA‐mediated invasion mechanisms in *Bactrocera dorsalis*.

Taken together, our study reveals a miRNA‐mediated post‐transcriptional mechanism governing thermal adaptation during *B. dorsalis* range expansion (Figure 6). Integrated analyses showed that invasive front populations display reduced phenotypic plasticity through genetic assimilation of frontloaded genes. Functional and evolutionary evidence identifies *thw* as a key cold‐tolerance gene, with its miR‐276b‐mediated regulation via 3’UTR targeting and conserved chitin‐binding domain constituting a critical adaptation axis. These findings highlighted the role of miRNA regulation of plasticity in species expansion responses to climate change.

## 3 Discussion

We compared the phenotypic responses and transcriptional plasticity to thermal acclimation, along with the regulatory role of miRNAs, in two populations of the invasive insect *B. dorsalis*: the southern ancestral and the northern derived population in China. Our findings revealed that the northern population exhibited a trend of reduced plasticity at both phenotypic and gene expression levels compared to the southern population. This pattern was consistent with genetic assimilation, in which genes that were constitutively upregulated (frontloaded) in the northern population required thermal acclimation in the southern population. miRNAs appeared to play a critical regulatory role in this divergence, with their expression evolution likely driving region‑specific gene expression profiles. Additionally, we identified a conserved thermal adaptation pathway in *B. dorsalis*, in which miR‐276b regulated cold tolerance by modulating *thw*, a hub gene in the cold‑tolerance network. This pathway likely contributes to the successful range expansion of *B. dorsalis* by enhancing its ability to cope with varying thermal environments. These results highlight the importance of miRNA‐mediated gene regulation in shaping phenotypic plasticity and adaptive responses during biological invasions.

### 3.1 Invasive Front Population Exhibits Reduced Phenotypic Plasticity

Contrary to the climatic variability hypothesis (CVH) which predicts greater plasticity in variable high‐latitude environments [73, 74], our findings demonstrate higher plasticity in the southern subtropical population across multiple traits: thermal tolerance, body size, and adult weight (Figure 1C–G). This pattern aligns with several empirical studies showing that tropical populations can exhibit higher thermal plasticity than their temperate counterparts, as demonstrated in *D. melanogaster* and *D. serrata* [75, 76]. Such patterns suggest that in stable environments, fitness‐related traits might maintain higher plasticity, potentially as an adaptive strategy to cope with occasional environmental fluctuations [77, 78]. The reduced plasticity observed in northern populations likely results from multiple interacting factors: (1) genetic bottlenecks during range expansion that reduce genetic variation underlying plastic responses [79]; (2) strong selection for canalized traits that enhance fitness in novel environments [78, 80]; and (3) trade‐offs between plasticity and basal thermal tolerance [81]. Indeed, the northern population's higher basal cold tolerance may represent an adaptive strategy compensating for reduced plasticity, a strategy previously noted in insects colonizing new environments [81].

The significant region × acclimation temperature interaction for cold tolerance (CT_min_) but not heat tolerance (CT_max_) (Table S2) suggests distinct evolutionary constraints on thermal adaptation. This pattern aligns with studies showing greater evolutionary lability of cold tolerance compared to heat tolerance in insects, a trend documented across ectotherms and within *Drosophila* [82, 83]. The northern population's improved cold tolerance may reflect ongoing adaptation to cooler climates at the range front [84], as observed in other invasive species such as *Corythucha ciliata* [85] and *Bemisia tabaci* [86], while conserved heat tolerance likely corresponds with relatively stable maximal temperatures at higher latitudes. Additionally, northern populations developed larger body size and greater adult dry weight after thermal acclimation, consistent with the results of Thompson et al. [14] Larger body size may provide advantages in flight capacity, energetic efficiency and thermal stress resistance [87, 88], supporting establishment in novel environments. Together, these findings suggest how invasive front populations can evolve reduced plasticity as a consequence of range expansion dynamics, while concurrently developing alternative adaptive strategies, such as increased constitutive tolerance and larger body size, to cope with novel environmental challenges.

### 3.2 Genetic Assimilation Drives Gene Expression Responses to Thermal Acclimation

Our study highlights the pivotal role of genetic assimilation in shaping gene expression plasticity during thermal acclimation in *B. dorsalis*. A substantial proportion (28.6%–51.2%) of genes showing evolved plasticity followed an assimilated pattern, indicating that temperature‐responsive traits have become constitutively expressed, thereby reducing reliance on plasticity. This suggests that selection has stabilized beneficial gene expression patterns in response to the more variable northern environment, minimizing the fitness costs associated with maintaining plasticity [89, 90]. In contrast, only a limited subset of DEGs (19.1%–37.1%) exhibited evolved plasticity, pointing to a limited genetic basis for plasticity [91, 92, 93]. This constraint may reflect either the scarcity of genetic variation or the high energetic costs of sustaining plastic responses under fluctuating conditions, highlighting the evolutionary trade‐offs in maintaining plasticity in variable environments. While plasticity can be beneficial in stable environments, it can be energetically costly and may reduce fitness when environmental conditions fluctuate unpredictably [91, 92]. In contrast, genetic assimilation allows populations to “canalize” beneficial traits, reducing the need for costly plastic responses, and enhancing survival in unpredictable environments [31]. This aligns with theoretical predictions and empirical studies that plasticity is often favored in stable environments, whereas genetic assimilation dominates in variable environments [29, 90], a trend observed in marine systems such as corals [30] and the seagrass [94] adapting to thermal stress.

The high proportion of genes exhibiting pre‐adapted (frontloaded) expression in northern populations further supports this hypothesis. Such pre‑activation likely enables rapid thermal responses without requiring substantial gene expression adjustments, enhancing energy efficiency and thermal tolerance in variable environments, albeit potentially at the cost of reduced body size. Similar trade‐offs have been documented in other systems, for example, in corals, the constitutive expression of heat shock proteins and antioxidants enhances thermal resilience but compromising growth and reproductive success [95, 96]. Thus, our findings suggest that in variable environments, the costs of maintaining plasticity may outweigh its benefits, favoring the stabilization of beneficial gene expression patterns through genetic assimilation [89, 97, 98].

Tissue‐specific analyses further elucidated the complex interplay between genetic assimilation and plasticity. Muscle exhibits a significantly higher proportion of genes associated with the evolution of expression plasticity compared to head, reflecting their distinct functional roles. Muscle may require greater transcriptional plasticity to maintain performance under fluctuating temperatures. In contrast, brain and sensory organs in head may rely more on canalized gene expression to ensure stable neural functions, while potentially regulating behavioral adaptations through more rapid, neuromodulatory pathways that are less dependent on broad transcriptional changes [99, 100]. This pattern is supported by findings in zebra finches *Taeniopygia guttata*, in which heat exposure caused significant gene expression changes in the testes, but few in the brain, indicating a weaker response in neural tissues [101]. In that system, behavior was linked to specific neuromodulatory pathways, a pattern consistent with the neural‐related functions identified in our head‐derived gene module associated with cold tolerance.

Together, these findings highlight the complex balance between adaptation, plasticity, and fitness costs, providing a mechanistic explanation for the poleward spread of *B. dorsalis*, and its ability to colonize new thermal niches. Our study underscores the importance of genetic assimilation as a driver of adaptive evolution, and offers new insights into the molecular basis of thermal adaptation in insects.

### 3.3 miRNAs Regulate Thermal Adaptation and Population Divergence during Range Expansion

MiRNAs have emerged as key regulators of evolutionary change by modulating gene expression and can facilitate rapid evolutionary adaptation during processes such as range expansion [102, 103]. In this study, we found that miRNAs play a critical role in regulating “frontloading” genes, which exhibit population‐specific expression patterns between southern (ancestral) and northern (invasive front) populations of *B. dorsalis* in response to thermal acclimation. This suggests miRNAs contribute to rapid phenotypic adaptation during range expansion by fine‐tuning gene expression networks [103]. The observed miRNA‐mediated regulatory patterns likely reflect a combination of environmental and evolutionary processes [104]. While genetic variation cannot be entirely ruled out, for example, *cis‐*regulatory genetic variant may underpin the regulation of genes responsive to thermal acclimation, which would require higher‐resolution techniques such as whole‐genome resequencing to identify the possible cause loci, and elucidate the causal relationships between miRNA regulation, gene expression, and phenotypic adaptation [105, 106]. Several lines of evidence suggest that miRNA‐mediated post‐transcriptional regulation plays a dominant role in shaping population divergence. Population genetic studies have found no significant genetic differentiation between these populations [64, 65, 66], and the relatively recent colonization history of *B. dorsalis* in China (southern populations: ∼1934; northern populations: post‐2000) [107, 108] makes it unlikely that genetic variation alone could account for the observed phenotypic differences. Instead, the significant overlap between DEGs and predicted miRNA targets (Figure S10) strongly supports the hypothesis that post‑transcriptional regulation by miRNAs contributes importantly to the observed phenotypic divergence.

A key finding of this study is the identification of a conserved miR‑276b‐*thw* regulatory axis. Functional validation confirmed that miR‑276b targets the 3’UTR of *thw* and that its knockdown of *thw* significantly alters cold tolerance. This pathway appears to be evolutionarily conserved, as miR‐276b has been implicated in cold adaptation in diverse insect species, including the Colorado potato beetle [109], the freeze‐tolerant *Eurosta solidaginis* [110, 111], and overwintering *Lissorhoptrus oryzophilus* [112]. Notably, the *thw* (*thawb*) gene, containing a conserved family 14 carbohydrate‐binding module (CBM_14; Pfam PF01607) known to specifically bind chitin and its oligomers [113], was initially identified in *Drosophila* through nociceptor‐specific RNAi screening where its knockdown impaired thermal nociception responses [114]. Subsequent evolutionary analysis revealed significant selection signals between climate‐adapted *Drosophila* populations [115], suggesting its adaptive role in thermal stress response. The CBM_14 domain, typically associated with chitinolytic enzymes or chitin‐binding lectins [113], may facilitate this function given chitin's critical structural roles in insect cuticles, trachea, and peritrophic matrices that mediate environmental stress protection [116]. These findings suggest that the miR‐276b‐*thw* regulatory axis represents a conserved mechanism for thermal adaptation, potentially contributing to the successful range expansion of *B. dorsalis*. However, we only validated the role of miR‐276b and *thw* gene at adult stage, while previous studies have demonstrated that chitin‐binding proteins and cuticle formation pathways are often regulated in a stage‐specific manner during insect development [117]. In addition, the regulatory role of miRNA could also differ between developmental stages in insects [118]. Thus, to distinguish between whether the responses of *thw* genes and the associated regulatory miRNA are induced by our experimental treatment or due to developmental plasticity, further studies are warranted to validate the miR‐276b‐*thw* axis across developmental stages.

Our results highlight the complex interplay between phenotypic plasticity, gene expression, and miRNA regulation in shaping thermal adaptation during range expansion (Figure 6). While the precise mechanisms linking miRNA‐mediated regulation to population divergence require further investigation, our findings suggest that miRNAs may facilitate rapid adaptation by modulating the expression of key stress‐responsive genes. It is important to note that our study focused on adaptation to long‐term, mild cold acclimation. The molecular pathways we identified under these conditions, while important for plasticity responses to temperature stress, may not be the only explanation for *B. dorsalis* to successfully overwinter (e.g., T99 ≈ −10°C). Current studies have suggested that long‐term acclimation and short‐term hardening under temperature stress may involve distinct biological pathways, and thus both are important to facilitate adaptive responses to future stress in insects [119, 120]. Thus, future research should aim to integrate our findings with investigations into acute extreme cold tolerance [121], and assess responses in populations from a wider and continuous latitudinal gradient to achieve a comprehensive understanding of the complex mechanisms enabling northward expansion. Finally, future studies could focus on cross‐generational plasticity and the stability of miRNA‐mediated regulatory networks to better understand their role in adaptive evolution [122, 123], and integrate the expression of miRNA‐related plasticity into a landscape transcriptome model to more accurately predict the responses of invasive species to future climates and establishment possibility [124].

## 4 Conclusion

Climate change drives the continuous shifts in species’ distribution ranges. To predict future invasions and understand how populations can evolve rapidly in response to novel environments, it is crucial to understand the mechanisms underlying adaptive plasticity. Our results support the hypothesis that populations at the invasion front adapt to thermal environments through reduced phenotypic and gene expression plasticity, thereby facilitating the expansion of their invasive range. Additionally, we highlight the potential role of miRNAs in adaptive evolution and colonization under changing environments. Through network analysis and molecular experiments, we identified a novel miRNA‐mediated pathway involved in insect thermal adaptation. Specifically, miR‐276b regulated the expression of *thw* gene to promote cold tolerance in *B. dorsalis*. Overall, our study emphasizes that effective miRNA‐mediated thermal adaptation strategies enable species to accelerate the adaptation process and expand their distribution range during invasions. This study represents a stride toward a more holistic understanding of the climatic adaptive potential in natural populations of invasive species.

## 5 Experimental Section

### 5.1 Sample Collection and Experimental Design

We collected *B. dorsalis* larva samples (F_0_) from wild infested fruits across four locations in China from June to October 2020 (Figure 1A). This paired‐edge sampling design included two southern‐edge populations (Danzhou, Hainan Province: DZ, 108.83472° E, 19.50881° N; Guangzhou, Guangdong Province: GZ, 113.36164° E, 23.15889° N), and two northern‐edge populations (Wuhan, Hubei Province: WH, 114.35794° E, 30.45174° N; Nanyang, Henan Province: NY, 111.84054° E, 32.9966° N). The southern sites represented ancestral invasive populations that first invaded China [125, 126], and the northern‐edge populations constituted derived populations that have recently colonized the northern range boundary and can successfully overwinter [107, 127]. Northern‐edge regions are characterized by lower annual mean temperature (AMT, 12.5°C–22°C) and higher climatic variability than southern‐edge regions (20.5°C–28.5°C) [128]. Approximately 50 individuals per location were transported to MARA Key Lab of Surveillance and Management for Plant Quarantine Pests, and acclimated at 25°C for one generation (F_1_) as described previously [129] to remove recent environmental effects on gene expression [130, 131, 132].

We then randomly exposed eggs (F_2_) from each population to warm (32°C), cold (17°C) or control (25°C) conditions, respectively, and reared them until sexual maturity (Figure 1B). These acclimation temperatures represent the local monthly average maximum and minimum temperatures during the species’ active growing season, respectively, and were selected to mimic chronic, sub‑lethal thermal acclimation while ensuring the completion of the *B. dorsalis* life cycle [128, 132]. Adults were maintained at a density of 200 per rearing cage (45 × 45 × 50 cm) at each treatment temperature. We sampled sexually matured adults for sequencing, and used the rest for phenotypic trait assays (see Section 5.2). Sampling and assays were conducted between 19:00 and 23:00 to avoid circadian variability effects. Sex was not considered a factor, as it has been reported not to affect thermal tolerance in Tephritid fruit flies [133, 134]. We randomly selected an equal number of sexually matured adults, dissected and pooled heads and thorax muscles (hereafter referred to as “muscles”), frozen them in liquid nitrogen, and stored them at −80°C prior to RNA extraction. As key tissues involved in environmental adaptation, we chose heads due to their role in behavioral responses to external environmental stimuli [135], and thorax muscles due to their role in insect energy supplies [136, 137].

### 5.2 Phenotypic Trait Assays

To understand how plastic responses to thermal acclimation varied among populations, we assessed patterns of plasticity in key phenotypic traits known to depend on temperature during development: critical thermal limit, adult size, and body mass. The phenotypic responses to thermal acclimation were similar between the two populations within each region (Figure S4). To enhance the robustness of our analysis, we combined the two populations within each region for downstream analyses, resulting in six groups: southern‐edge region under 17°C (S_Cold), 25°C (S_CK), and 32°C (S_Warm); and northern‐edge region under 17°C (N_Cold), 25°C (N_CK), and 32°C (N_Warm).

#### 5.2.1 Critical Thermal Limit

We used a dynamic temperature ramping protocol to assess CT_min_ and CT_max_, defined as the loss of muscular coordination, and consequently losing the ability to respond to mild stimuli [138]. Following Nyamukondiwa and Terblanche [136] with minor modifications, 30 flies per population/acclimation group were randomly and individually placed in 4 ml Eppendorf tubes connected to a water‐filled double‐jacketed chamber linked to a programmable water bath (SCIENTZ, DC‐4010). Chamber temperature was recorded with a thermocouple and digital thermometer (Grows Instrument, HB6801). Assays began at the control temperature (25°C), with heating (CT_max_) and cooling (CT_min_) rates at 0.1°C/min. Flies were stimulated every minute, and CT_max_ and CT_min_ were recorded upon loss of response.

#### 5.2.2 Adult Size

We estimated body size using wing area and femur length, following the temperature‐size rule common in ectotherms [77, 139]. Thirty adult flies from each treatment were preserved in a 70% ethanol/30% glycerol solution (SH solution) and dissected. Left first legs and left wings were mounted on microscope slides in SH solution and photographed with a Leica M80 stereo microscope (Leica, Heerbrugg, Switzerland). All images were analyzed using ImageJ. Femur length was estimated between the junctions of the femur with the coxa and tibia, following Chakraborty et al. [77] (Figure S1A). Wing area was estimated according to Yu et al. [140] (Figure S1B,C). The forewings of flies were scanned at 600 dpi with an Epson photo scanner (Epson V550, Batam, Indonesia), and converted to black‐white bitmaps at 600 dpi using Adobe Photoshop CS2 v9.0 (Adobe, San Jose, CA, USA; https://www.adobe.com/products/photoshop.html). We then used an M‐file in MATLAB v2009a (MathWorks, Natick, MA, USA; https://www.mathworks.com/products/matlab.html) developed by Shi et al. [141] to extract the planar coordinates of the wing outlines. The R script (v3.6.1 R Core Team, 2019) developed by Su et al. [142] was used to calculate wing area, length, and width. Wing length was defined as the maximum distance from base to tip, and width as the maximum distance of any two points on the wing's boundary perpendicular to the wing length axis.

#### 5.2.3 Body Mass

Adult dry mass was measured as a proxy for body mass, a trait linked to fitness, and invasion potential [143, 144, 145]. After reaching sexual maturity, 150 adults per treatment were randomly collected, dried at 60°C for 48 h, and weighed.

### 5.3 RNA Extraction, mRNA Sequencing and miRNA Sequencing

We performed three biological replicates per treatment, each with 5 males and 5 females, except for the GZ population under cold acclimation (only two replicates due to limited samples), totaling 70 samples. We first homogenized tissues using a homogenizer (DHFSTPRP‐24/48) with Zirconia/Silica beads (1 min, 6 m/s), and extracted total RNA from tissue homogenate using the TRIzol reagent kit (15596‐018, Invitrogen, USA). RNA quantity and quality were assessed using the Nanodrop 8000 spectrophotometer (Thermo Fisher, USA) and the Agilent 2100 Bioanalyzer (Agilent Technologies, USA). mRNA‐seq libraries were created using the NEBNext Ultra Directional RNA Library Prep Kit (E7420, NEB, USA) by the Novogene Bioinformatics Technology Co. Ltd. (Tianjin, China), with quality checked using the Bioanalyzer traces (Agilent Technologies, USA). The libraries were sequenced on the Illumina NovaSeq 6000 platform (Novogene) with 150‐bp paired‐end reads.

For miRNA sequencing, RNA was extracted from 70 tissue homogenates using the miRNeasy mini kits (74106, Qiagen, Germany), and qualified as above. The qualified RNA was submitted to the BGI Biotech Co., Ltd. (Wuhan, China) for small RNA library preparation using the MGIEasy Small RNA Library Prep Kit (940‐200026‐00, MGI, China). Library quality and quantity were examined by the Agilent 2100 Bioanalyzer and the ABI StepOnePlus Real‐Time PCR System (ABI, USA), and sequencing was performed on the MGI2000 platform (BGI) with 50‐bp single‐end reads.

### 5.4 mRNA Differential Expression Analysis

We used FASTQC v0.11.8 (http://www.bioinformatics.babraham.ac.uk/projects/fastqc/) to check the quality of raw reads, and Trim Galore! v0.6.6 (www.bioinformatics.babraham.ac.uk/projects/trim_galore/) to remove adapter sequences and trim low‐quality reads. Cleaned reads were re‐evaluated with FASTQC and mapped to the *B. dorsalis* genome (NCBI Assembly accession ASM78921v2) using HISAT2 v2.1.0 [146] with the parameters: –new‐summary ‐k 1 –rna‐strandness RF. We then used SAMtools v1.3.1 [147] to sort mapped reads based on reference coordinates. PCR duplicates were removed using the MarkDuplicates function in Picard v2.25.1 (http://broadinstitute.github.io/picard/). We quantified read count for each gene using HTSeq‐Count v0.12.4 [148] with the parameters: ‐f bam ‐s reverse ‐r name ‐t exon ‐union. Genes with < 1 read per million in at least half of the 70 samples were filtered out to avoid noise from weakly expressed genes [149]. We then normalized the relative expression levels by the TMM method in edgeR v3.34.1 [150] with default settings.

The PCA of 9645 expressed genes using the R package PCAtools v2.4.0 [151] confirmed clear separation between head and muscle samples (Figure S5A). We therefore performed differential expression analysis separately for each tissue using the R package DESeq2 v1.32.0 [152]. To make our analysis robust, two populations from the same region were pooled, resulting in six groups: southern‐edge under warm (S_Warm), cold (S_Cold), and control (S_CK); and corresponding northern‐edge groups (N_Warm, N_Cold, N_CK). We used negative binomial generalized linear models to test the effects of region, thermal acclimation, and interactions between these factors on each gene. Transcripts with differing responses to thermal acclimation across regions were identified using factors “Region” (levels = southern‐edge and northern‐edge) and “Thermal acclimation” (levels = warm acclimation, cold acclimation, and control treatment), with an interaction model design = ∼ Region + Thermal acclimation + Region × Thermal acclimation. Seven pairwise comparisons were conducted to investigate gene expression differences: (i) S_Cold vs. S_CK; (ii) N_Cold vs. N_CK; (iii) S_Warm vs. S_CK; (iv) N_Warm vs. N_CK; (v) N_Cold vs. S_Cold; (vi) N_Warm vs. S_Warm; and (vii) N_CK vs. S_CK. These comparisons were also used for reaction norm pattern analysis (see Section 5.5). Genes with a log2‐fold change > 1 and a false discovery rate (FDR)‐adjusted *p*‐value (*p*_adj_) < 0.05 were considered differentially expressed. Shared or unique DEGs across regions, thermal acclimation conditions, and their interaction were summarized using the R package VennDiagram [153]. We further visualized the number of up‐ and downregulated DEGs and their overlap between regions.

### 5.5 Evolution of Expression Plasticity Analysis

Genes exhibiting evolved expression plasticity were defined as those showing a significant Region × Thermal acclimation interaction, indicating that the direction or magnitude of thermal response differed between populations. Following Renn and Schumer [44], we further classified these genes into four evolutionary patterns based on post‑hoc acclimation effects within each region (*p* < 0.05; Figure S2): (i) Assimilated: ancestral populations in southern region exhibit plasticity, but plasticity is lost in derived populations in northern region; (ii) Accommodated: the degree, but not the direction, of plasticity changes in derived populations compared to ancestral populations; (iii) Evolved plastic: plasticity is absent in ancestral populations but emerges in derived populations; (iv) Reversed: plasticity directions are opposite between populations. Remaining genes with a significant main interaction effect but no significant post hoc thermal acclimation differences were categorized as unclassified. In addition, we further identified “frontloaded” genes by adapting the method from Barshis et al. [30] In this study, frontloading refers to genes showing higher baseline expression in northern populations under control conditions, and are subsequently upregulated in southern populations after thermal acclimation, reflecting pre‐activation in the more cold‑tolerant northern populations before thermal stress.

### 5.6 Weighted Gene Co‐Expression Network Analysis and Enrichment Analysis

We used the R package WGCNA [46] to identify modules of co‐expressed genes associated with region, thermal acclimation or phenotypic traits. After generating modules, we calculated “eigengenes” representing the expression patterns of each module and correlated them with additional traits. Variance‐stabilized counts were filtered for outliers using the goodSampleGenes function, and a similarity matrix was constructed using Pearson correlation. Soft‐thresholding powers of 12 (head) and 5 (muscle) were selected to achieve scale‐free topology (Figure S6). Modules were identified by hierarchical clustering of topological overlap (minimum module size = 30), and similar modules were merged using Dynamic Tree Cut (threshold = 0.25). Finally, we correlated the expression of each module “eigengene” against a binary matrix containing categorical treatment information, with modules showing *p* < 0.05 considered treatments‑associated. Treatments included region (northern‐edge region: 1, southern‐edge region: 0), Normal (control temperature: 1, others: 0), Warm (warm acclimation: 1, others: 0), Cold (cold acclimation: 1, others: 0), and phenotypic trait data from section 5.2 (CT_min_, CT_max_, femur length, wing area, and adult weight). We focused on modules significantly correlated (*p* < 0.05) with CT_min_ (correlation coefficient < −0.5) or CT_max_ (correlation coefficient > 0.5), as these traits are crucial for assessing thermal tolerance. A negative correlation with CT_min_ indicates an association with greater cold tolerance. We also calculated GS and MM to measure the association between genes and modules. Hub genes were identified as those with MM > 0.8 and GS > 0.8. Network of hub genes was visualized using Cytoscape v3.7 (http://www.cytoscape.org/) [154].

For functional annotation, GO terms for all protein‐coding genes in the *B. dorsalis* genome were obtained using eggNOG‐mapper [155]. Enrichment analysis on gene sets from WGCNA modules and reaction‑norm categories was performed with the R package clusterProfiler v4.0.5 [156], using a *q*‐value cut‐off of 0.05.

### 5.7 miRNA Read Analysis and miRNAs Discovery

Small RNA reads were processed with Trim Galore! with parameters “trim_galore ‐a AGTCGGAGGCCAAGCGGTCTTAGGAAGACAA –length 18 –max_length 24 –stringency 3 –phred64” to remove adapters and retain sequences 18‐24 nt in length. Quality was assessed both before and after trimming using FASTQC. The processed reads were collapsed with the mapper.pl module of mirDeep2 v2.0.0.8 [157], and mapped to the *B. dorsalis* genome (NCBI Assembly accession ASM78921v2) using Bowtie v1.3.0 [158] with parameters “bowtie ‐f ‐n 1 ‐l 8 ‐a ‐m 5 –best ‐strata ‐al” [159], allowing one base mismatch, and reporting only the best alignment per read. Besides, we examined the read distribution among: i) known *B. dorsalis* miRNA hairpins from miRBase 22.1 (bowtie ‐f ‐v 0 ‐p 10); ii) ncRNA sequences from Rfam v14.5 (bowtie ‐f ‐v 2 ‐p 10 ‐a –best –strata); and iii) genomic features of the *B. dorsalis* assembly, including repeating (extracted with BEDtools v2.30.0 [160]), exons (seqkit [161]), and introns (custom Perl script; bowtie ‐f ‐v 0 ‐p 10). Overlapping assignments were resolved by priority using a custom Perl script: known miRNAs > ncRNA > repeat > exon > intron; remaining reads were defined as “unknown”.

For miRNA discovery, we used mapper.pl (‐j ‐r 5) to obtain position files for “unknown”, “intron”, and “known miRNAs”. We then used the miRDeep2.pl to identify miRNAs, using RNAfold to predict and fold potential pre‐miRNA precursor sequences [162], and randfold to assess their stability [163]. To improve prediction accuracy, we included known *B. dorsalis* miRNAs and Hexapoda miRNAs from miRBase as “related species” set [164]. Known miRNAs were defined by matches to miRbase, and the remainder were regarded as “novel miRNAs”. We retained predicted pre‐miRNAs with miRDeep2 score > 10, mature/star sequence lengths 20–26 nt, and significant randfold *p*‐value. Additionally, we applied additional stringent filters to reduce false positives [165]: ≥ 16‐nt arm complementarity, loop ≥ 8 nt, and a 2‐nt 3’ overhang on both mature and star strands. Genomic positions of miRNAs were determined by intersecting coordinates with the *B. dorsalis* annotation using BEDtools, with priority of 5’ UTR > 3’ UTR > exon > intron > intergenic regions.

### 5.8 miRNA Differential Expression Analysis

We mapped the trimmed reads to the *B. dorsalis* genome, then used the quantifier.pl module in miRDeep2 to quantify miRNA expression. We filtered the weakly expressed miRNAs as described for RNA‐seq data. The PCA of the 81 remaining miRNAs revealed two muscle samples from the NY population reared at 25°C (Bd25NY_M2 and Bd25NY_M3) as outliers (Figure S3). These were excluded from further analyses. PCA further showed clear separation between the two tissues (Figure S5B), and differential analysis was therefore conducted separately for each tissue. Differentially expressed miRNAs were analyzed using DESeq2 with the same seven contrasts as the mRNA data. miRNAs with a *p*_adj_ < 0.05 were considered significantly differentially expressed. We generated Venn diagrams to identify shared and unique miRNA responses between regions.

### 5.9 miRNA Target Prediction and Construction of miRNA‐mRNA Networks

We used three software packages to predict miRNA targets: RNAhybrid v2.1.2 [166] with parameters “‐b 10 ‐f 2,8 ‐m 10000 ‐v 3 ‐u 3 ‐s 3utr_fly ‐p 1 ‐e ‐20”, miRanda v3.3a [167] with settings “‐en ‐10” and strict mode, and TargetScan v7.0 [71] with default parameters. Only genes predicted by all three methods were considered further.

Although positive miRNA/gene target pairs correlations can occur in complex circuits, miRNAs are primarily understood as post‑transcriptional repressors [168, 169]. Therefore, this study focused on negatively correlated miRNA/gene pairs. We then intersected DEGs with predicted miRNA targets, and calculated Pearson correlations between differentially expressed miRNAs and their candidate targets using mirlab [170]. Pairs showing significant inverse expression (negative correlation) were used to construct miRNA‑mRNA regulatory networks in Cytoscape, with particular attention to genes involved in expression plasticity (Section 5.5) and hub genes from co‑expression modules (Section 5.6).

### 5.10 Expression Analysis of miR‐276b and its Target Gene *thw*

We selected miR‐276b for functional analyses because it was the only miRNA differentially expressed under both warm and cold acclimation in both populations (Figure 4A), indicating a potential broad role in thermal adaptation. Its predicted target, *thw*, was selected as the central hub gene associated with thermal tolerance (Figure 3E), and it showed cold response in both populations (Figure 3F). For functional assays, sexually mature adults were injected into the ventral abdomen with 40 pmol of miR‐276b mimic (GeneCreate, Wuhan, China) using a Nanoject II microinjection device (Drummond Scientific, USA). An equivalent dose of mimic‐NC was injected as a control (sequences in Table S5). After 24 h, thorax muscles from two adults were pooled as one biological replicate to evaluate relative expression of *thw* using quantitative real‐time PCR (qRT‐PCR). Primers are listed in Table S6, with efficiency validation shown in Figure S14. The small noncoding RNA U6 and 18s ribosomal RNA (*18s*) were used as reference genes to normalize miRNAs and mRNA expression, respectively.

### 5.11 In Vitro Dual Luciferase Reporter (DLR) Gene Assays

Targetscan was first used to predict target sites between the target gene *thw* and miR‐276b. A ∼200 bp 3’ UTR with predicted miR‐276b target sites in *thw* were cloned into the pmirGLO vector (E1330, Promega, USA), downstream of the luciferase gene (creating pmirGLO‐*thw*constructs) using SacI and XhoI restriction sites, and verified by sequencing. A mutant sequence for *thw* was generated with the QuickMutation kit (D0219M, Beyotime, China). HEK293T cells seeded in 24‐well plates were co‐transfected with the luciferase reporter plasmid (WT or MT) and mimic‐276b using Lipofectamine 2000 (Invitrogen), with mimic‐NC as a negative control. After 48 h, luciferase activity was determined by GeneCreate Biotechnology Co., Ltd (Wuhan, China) using the Dual‐Luciferase Reporter Assay system (E1910, Promega, USA) and Infinite F200 (TECAN, Switzerland). Normalized luciferase activity was calculated as the ratio of Renilla to firefly luciferase activity. Each experiment included five biological replicates.

### 5.12 Evolutionary Analysis and Functional Validation of *thw* and miR‐276b in *B. Dorsalis*

For evolutionary analysis, we first identified functional domains of the THW protein using Constraint‐based Multiple Alignment Tool (COBALT; https://www.ncbi.nlm.nih.gov/tools/cobalt/) and InterPro (https://www.ebi.ac.uk/interpro/). Homologous sequences from 16 insect species were retrieved from NCBI, and analyzed using MEGA v12 with the neighbor‐joining method (1000 bootstrap replicates). Sequence conservation of the CBM_14 domain was visualized using WebLogo (http://weblogo.berkeley.edu/), and structural comparison with Popillia japonica's chitin‐binding Peritrophin‐A domain was performed using ESPript v3.0 (https://espript.ibcp.fr/ESPript/ESPript/index.php).

For functional validation, sexually mature adults were injected intra‐abdominally with 40 pmol of mimic‐276b or mimic‐NC. After 24 h, 30 individuals were randomly selected to detect CT_min_ or CT_max_ as described in Section 5.2. For RNAi, dsRNA (ds*thw* and dsGFP) was synthesized in vitro using the T7 RiboMAX Express RNAi System (P1700, Promega, USA). Primers for dsRNA synthesis are listed in Table S7. Adults were injected with 1 µg of dsRNA, and after 24 h, CT_min_ or CT_max_ were detected in 30 individuals per treatment. To verify knockdown, thorax muscles from two adults were pooled as one biological replicate to assess *thw* expression by qRT‐PCR, with four biological replicates per condition.

### 5.13 Statistical Analysis

The statistical analysis method used for each dataset was detailed in the corresponding figure legend. All experimental data were presented as mean ± standard error of the mean (SEM). A *p*‐value < 0.05 was considered statistically significant, and exact *p*‐values were reported where applicable (Dataset S10). Gene and miRNA expression levels from qRT‐PCR were analyzed using the 2^‐ΔΔCt^ method [171]. No‐template controls were included in all reactions to ensure specificity. All analyses were performed using R software v3.6.1 and IBM SPSS Statistics v26.0 software (SPSS Inc).

## Author Contributions

Z.L., Y.Z., and J.H. conceived the study. Y.Z., S.G., Y.H., and Z.L. sampled and reared the insects. Y.Z. performed the experiments and generated and analyzed the sequencing data. The manuscript was written by Y.Z. and J.H., with input from Z.L., J.D., S.G., and Y.H. The study was supervised by Z.L.

## Conflicts of Interest

The authors declare no conflicts of interest.

## Supporting information




**Supporting File**: advs74381‐sup‐0001‐SuppMat.docx.

**Supporting File**: advs74381‐sup‐0002‐Data.zip.

## Data Availability

RNA‐seq and miRNA‐seq sequence reads are deposited in the NCBI Sequence Read Archive under BioProject PRJNA1039146 and PRJNA1037329. All scripts associated with the analysis are available in Dataset S11.
