# Supplementary material for: Thermal Plasticity is Regulated by a Key MicroRNA During Range Expansion of an Invasive Fruit Fly
Source: Adv Sci (Weinh). 2026 Feb 24;13(23):e07662. doi: 10.1002/advs.202507662 (PMC13104144; doi:10.1002/advs.202507662)
Supplement: Supplementary file 1 — Supporting File: advs74381‐sup‐0001‐SuppMat.docx. [file ADVS-13-e07662-s002.docx]

**
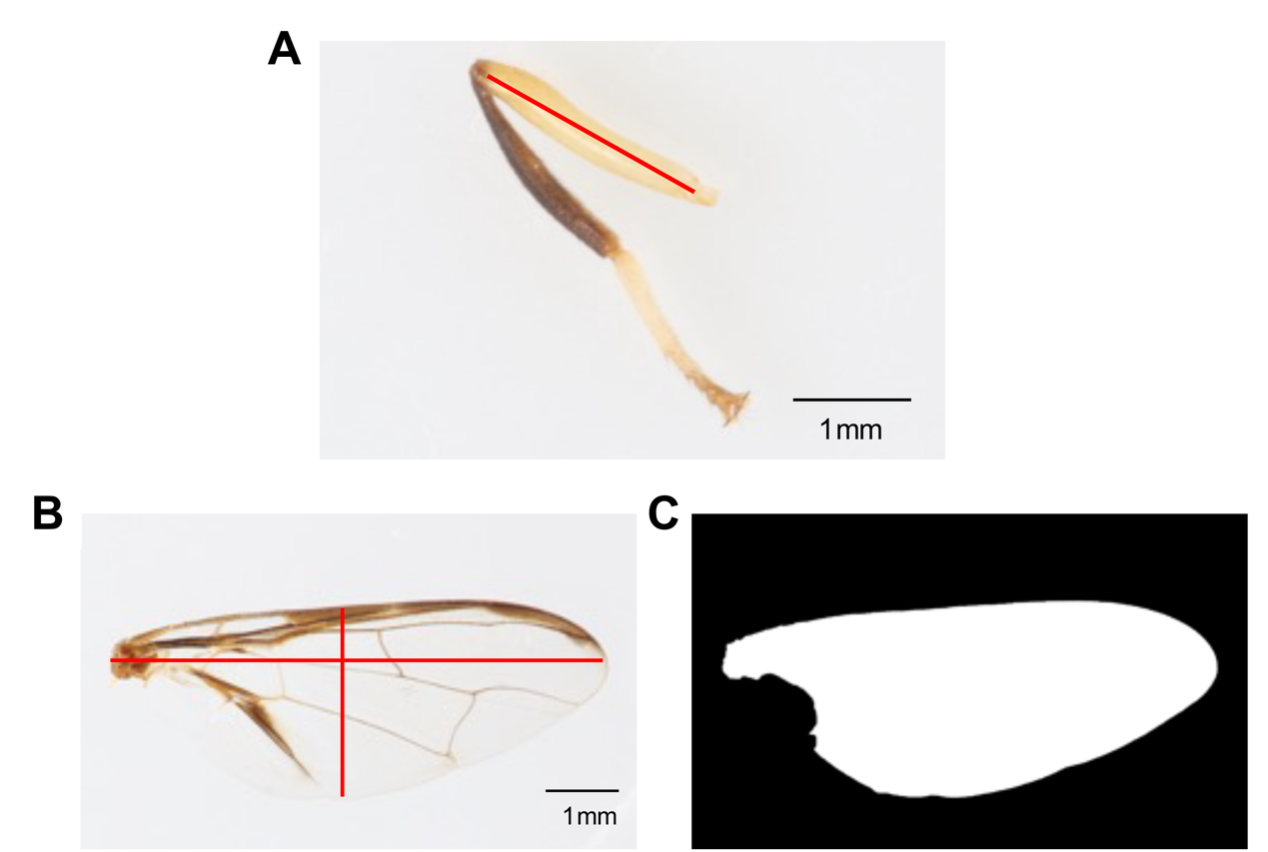
**

**Figure S1.** Methods for phenotypic indicators determinations in *Bactrocera dorsalis*.

Note: (**A**) femur length; (**B-C**) wing area

**
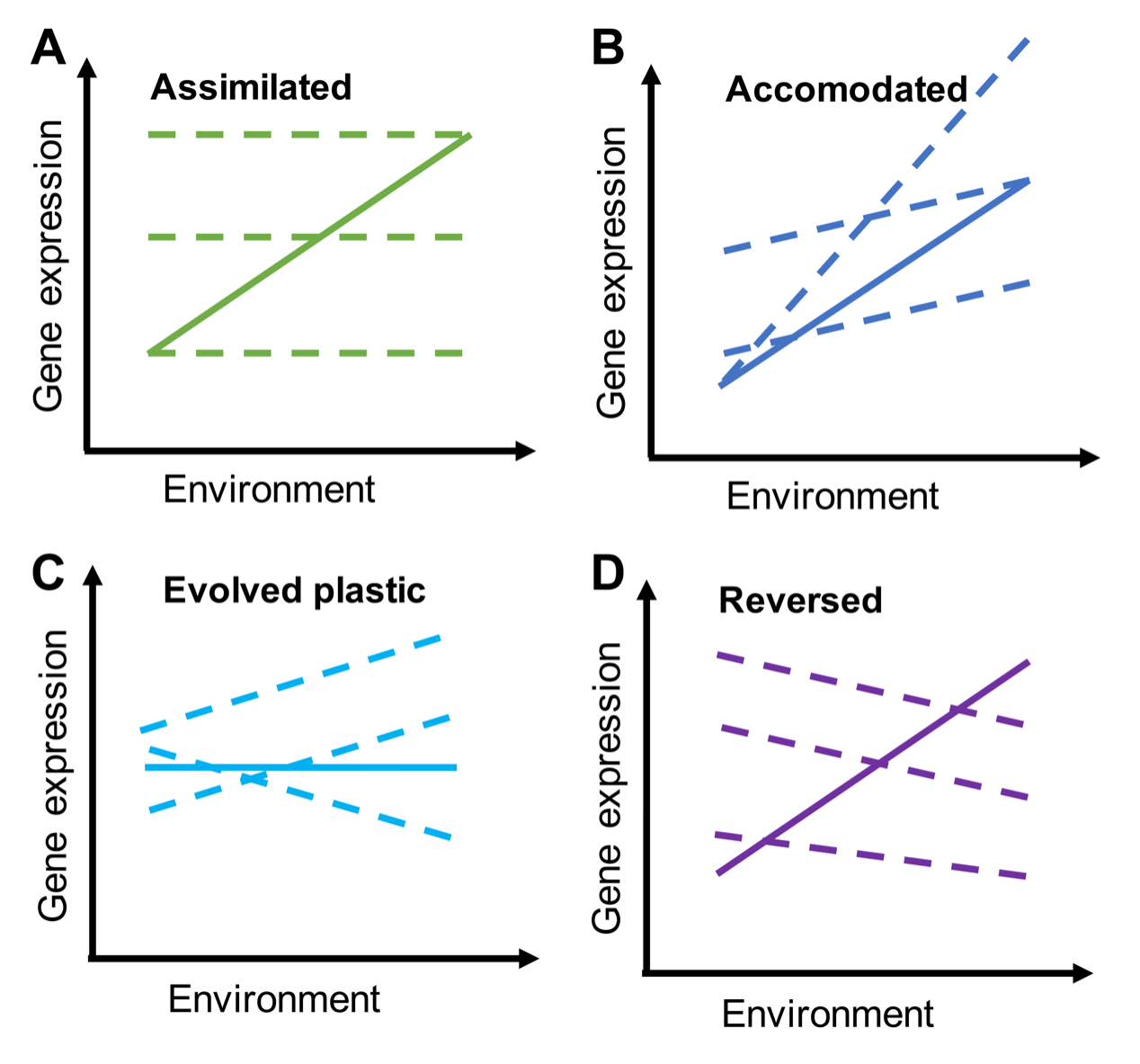
**

**Figure S2.** Norms of reaction for gene expression in the ancestral populations, i.e. southern-edge region (solid line) and derived populations, i.e. northern-edge region (dashed lines) based on Renn and Schumer (2013). (**A**) Assimilated: These genes are environmentally insensitive in the derived population, fixed at one extreme, or mid-level compared to the plastic ancestral expression. (**B**) Accommodated: These genes are environmentally responsive in both genotypes. (**C**) Evolved plasticity: These genes are sensitive to environment only in the derived genotype and not in the ancestral phenotype. (**D**) Reversed: The level of expression of these genes is sensitive to the environment in both genotypes, but the direction of regulation is reversed.

**
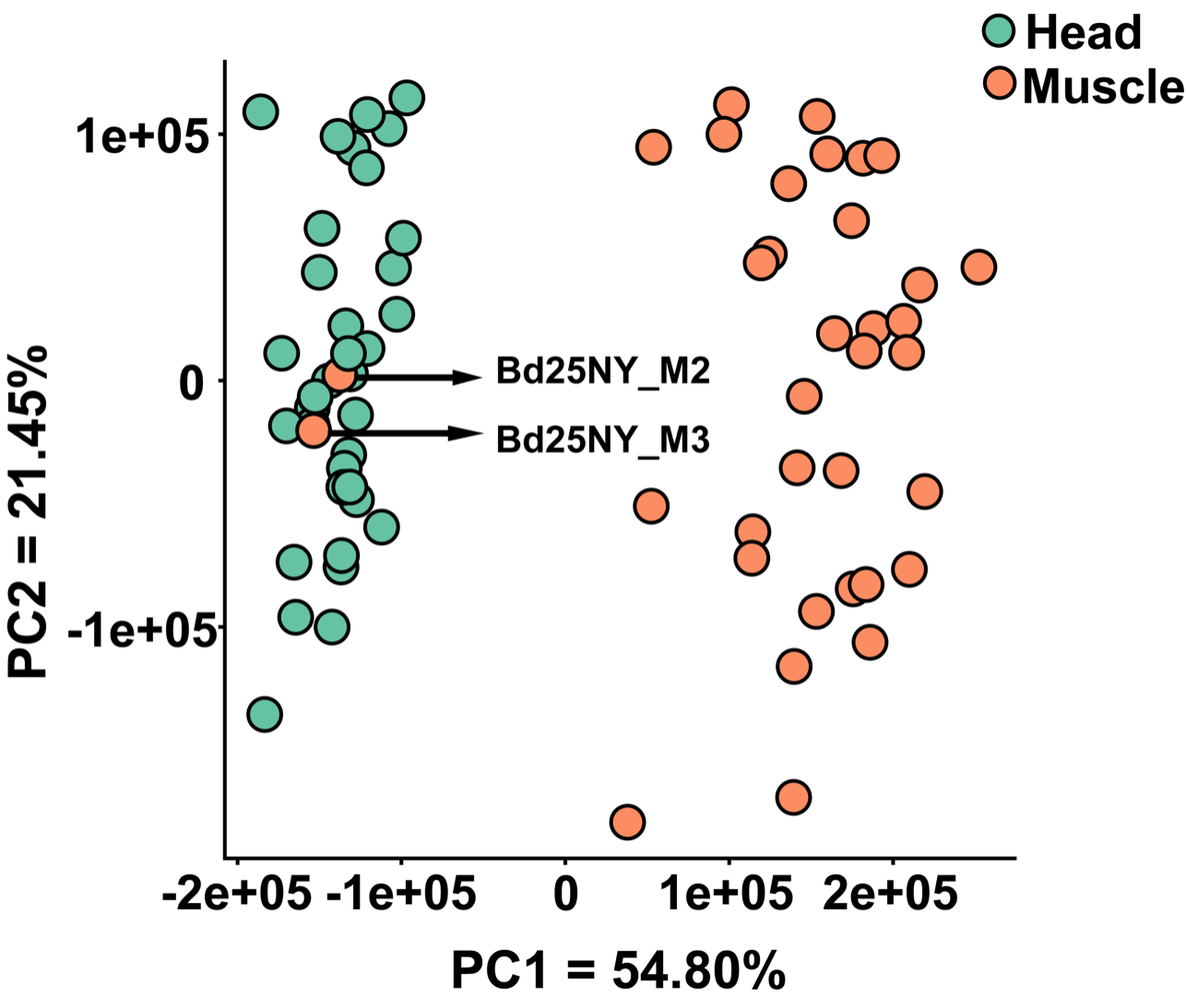
**

**Figure S3.** PCA of miRNA count data of samples including outlier sample (Bd25NY_M2 and Bd25NY_M3).

**
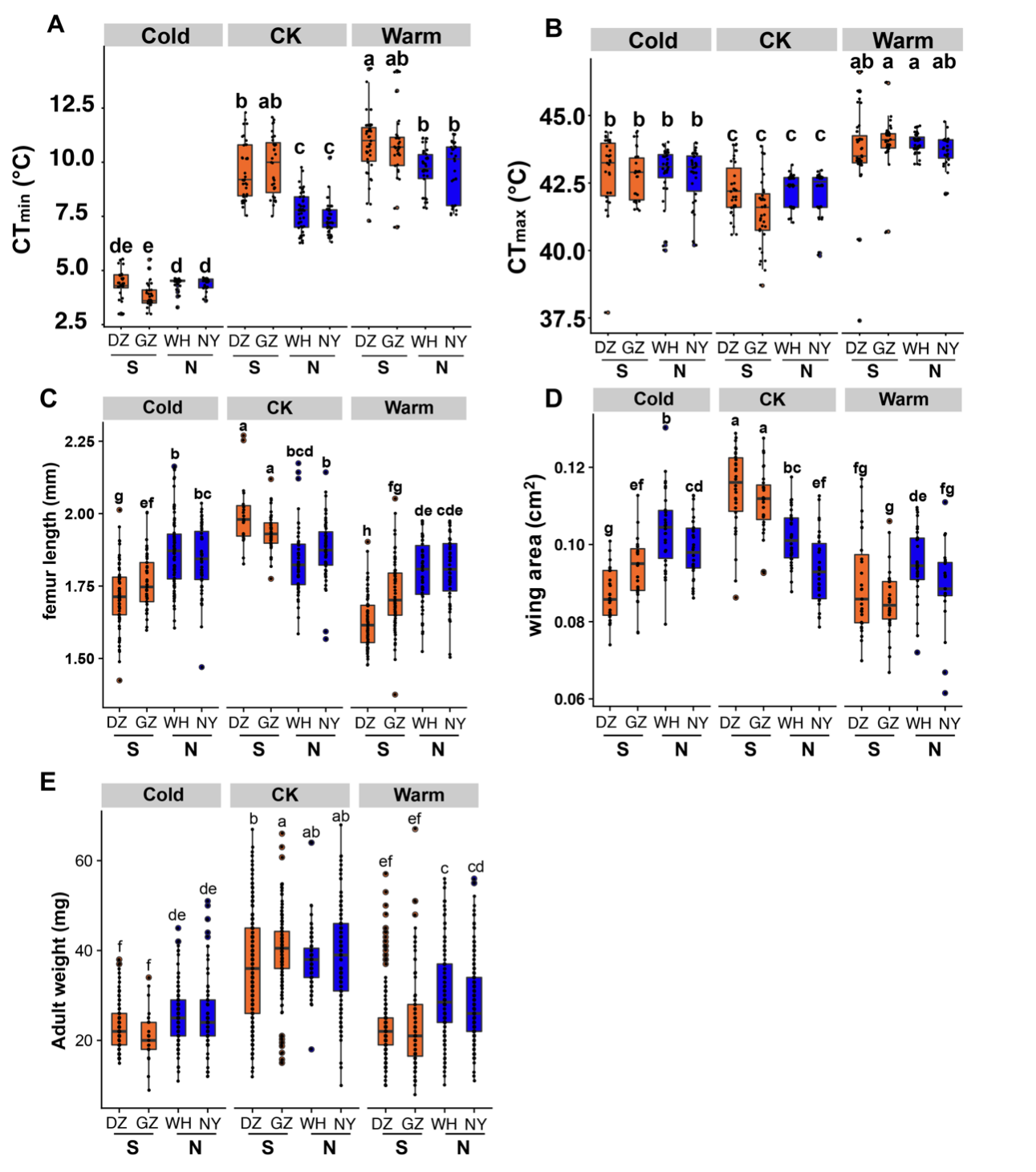
**

**Figure S4.** (**A**) CT_min_, (**B**) CT_max_, (**C**) femur length, (**D**) wing area, and (**E**) adult weight of *B. dorsalis* across populations after acclimation for one generation at cold, warm, and control temperatures. Vertical bars denote 95% confidence limits. A one-way ANOVA test was conducted separately for each population across temperatures. Different letters indicate significant differences at *p* < 0.05, and the same letters indicate no significant differences.

**
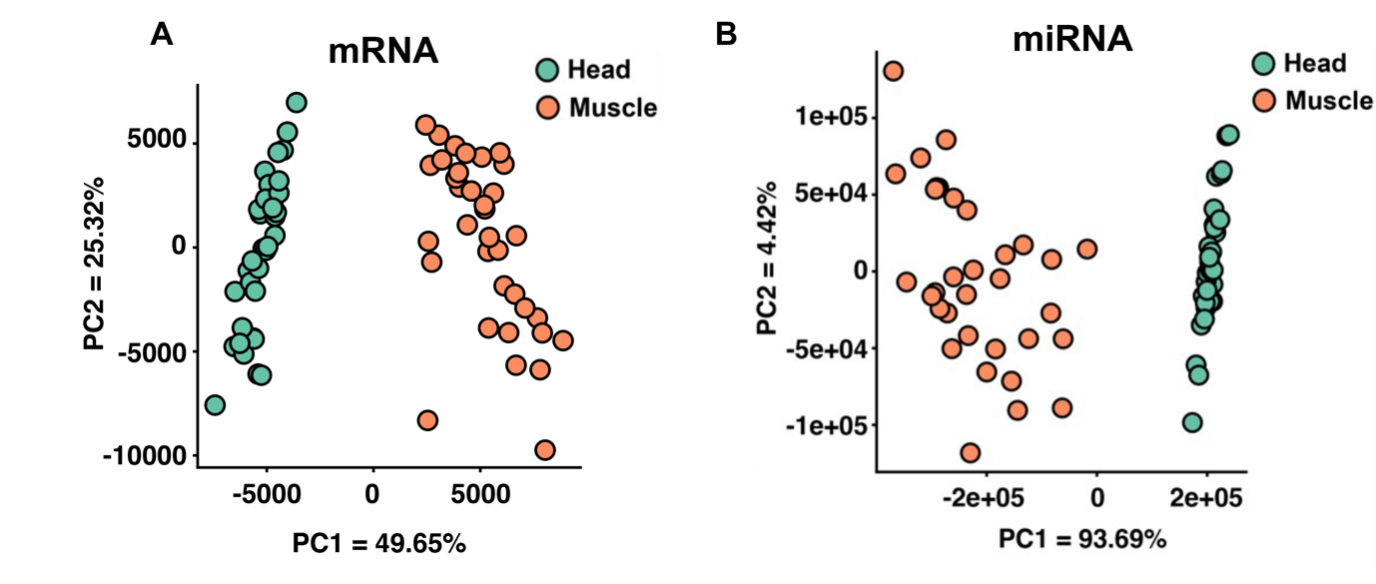
**

**Figure S5.** Principal component analysis (PCA) plots of (**A**) mRNA and (**B**) miRNA expression in head and muscle tissues of *Bactrocera dorsalis*.

**
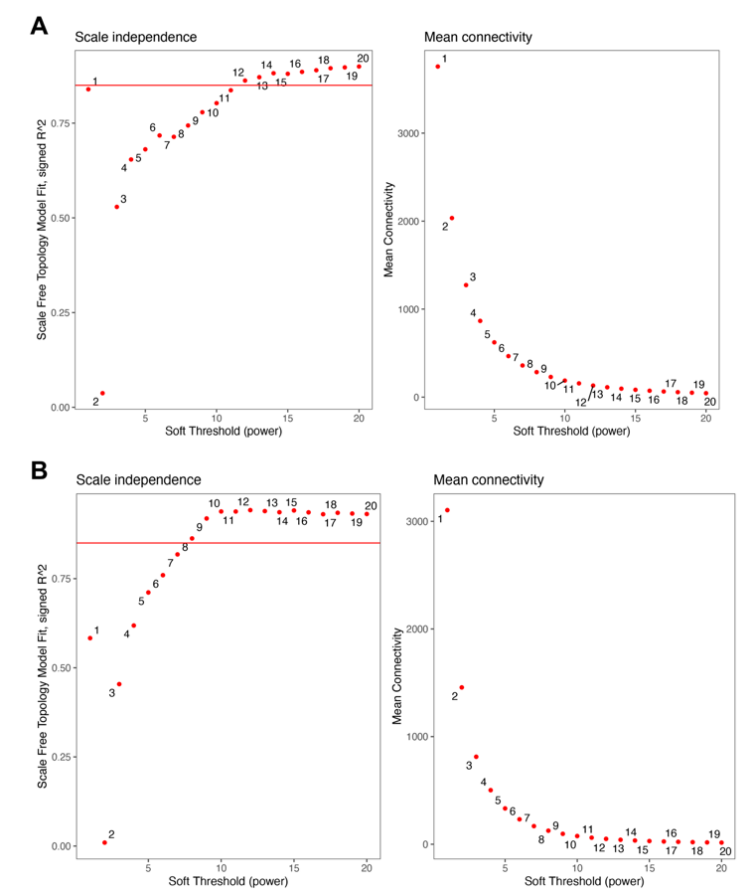
**

**Figure S6.** Network topology analysis for various soft-thresholding power levels in WGCNA for (**A**) head and (**B**) muscle tissues. The left panel shows the scale-free topology model fit (SFTMF, signed R^2^) as a function of the soft-thresholding power (STP). The right panel shows the mean connectivity (MC) as a function of STP.

**
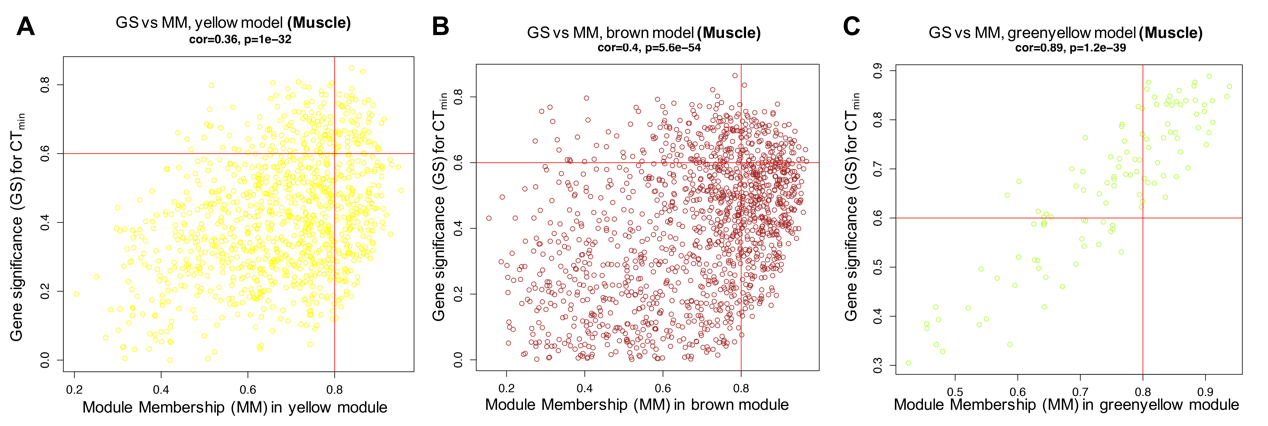
**

**Figure S7.** Scatterplots showing gene significance (GS) for CT_min_ versus module membership (MM) in the (**A**) yellow, (**B**) brown, and (**C**) greenyellow eigengene modules.

**
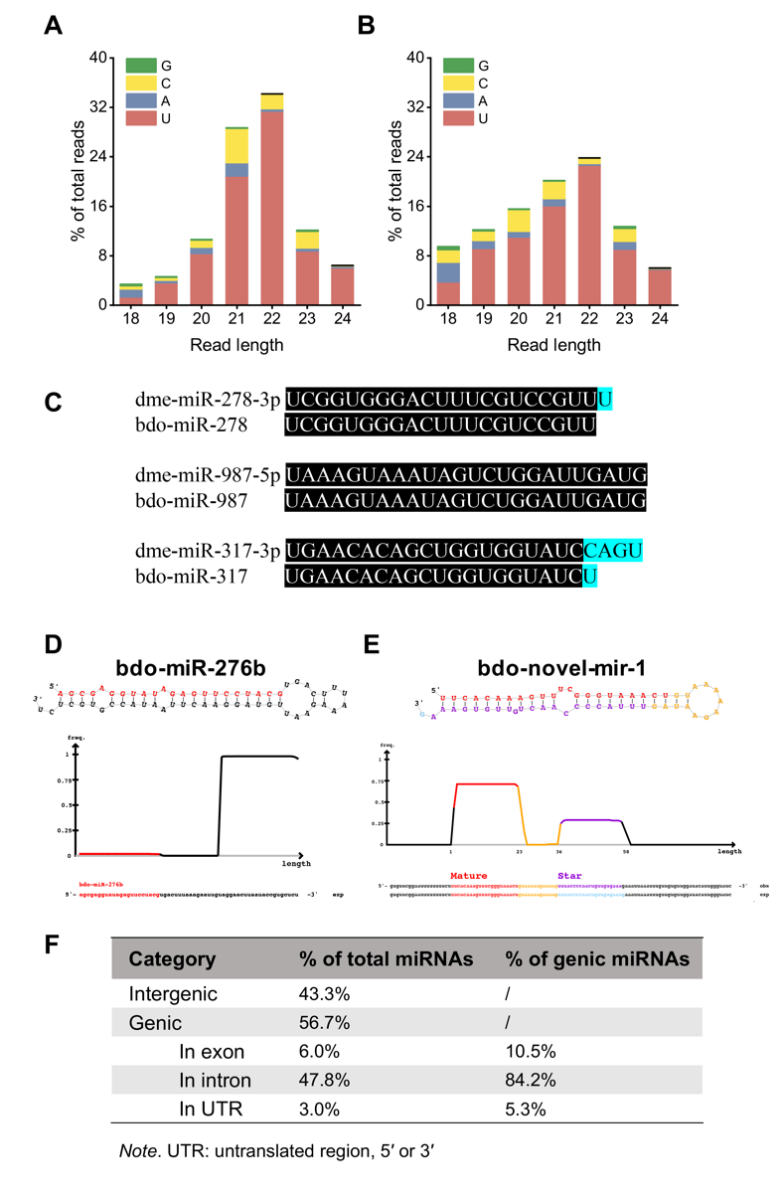
**

**Figure S8.** Sequence length distribution of pooled sequencing reads from *Bactrocera dorsalis* small RNA libraries, showing the proportion of reads starting with A, C, G or U in (**A**) head and (**B**) muscle tissues. The small peak at 22nt corresponds to the expected length of miRNAs. (**C**) Identification of three known miRNAs compared with other species. **(D-E)** Predicted precursor stem-loop structures of novel miRNAs, showing the mature strand (red), hairpin loop (yellow), and star strand (blue). The line graph shows read mapping frequency along different parts of the precursor sequence. Numbers above the line represent the number of mapped reads. (**F**) Genomic locations of identified miRNAs in *B. dorsalis*.

**
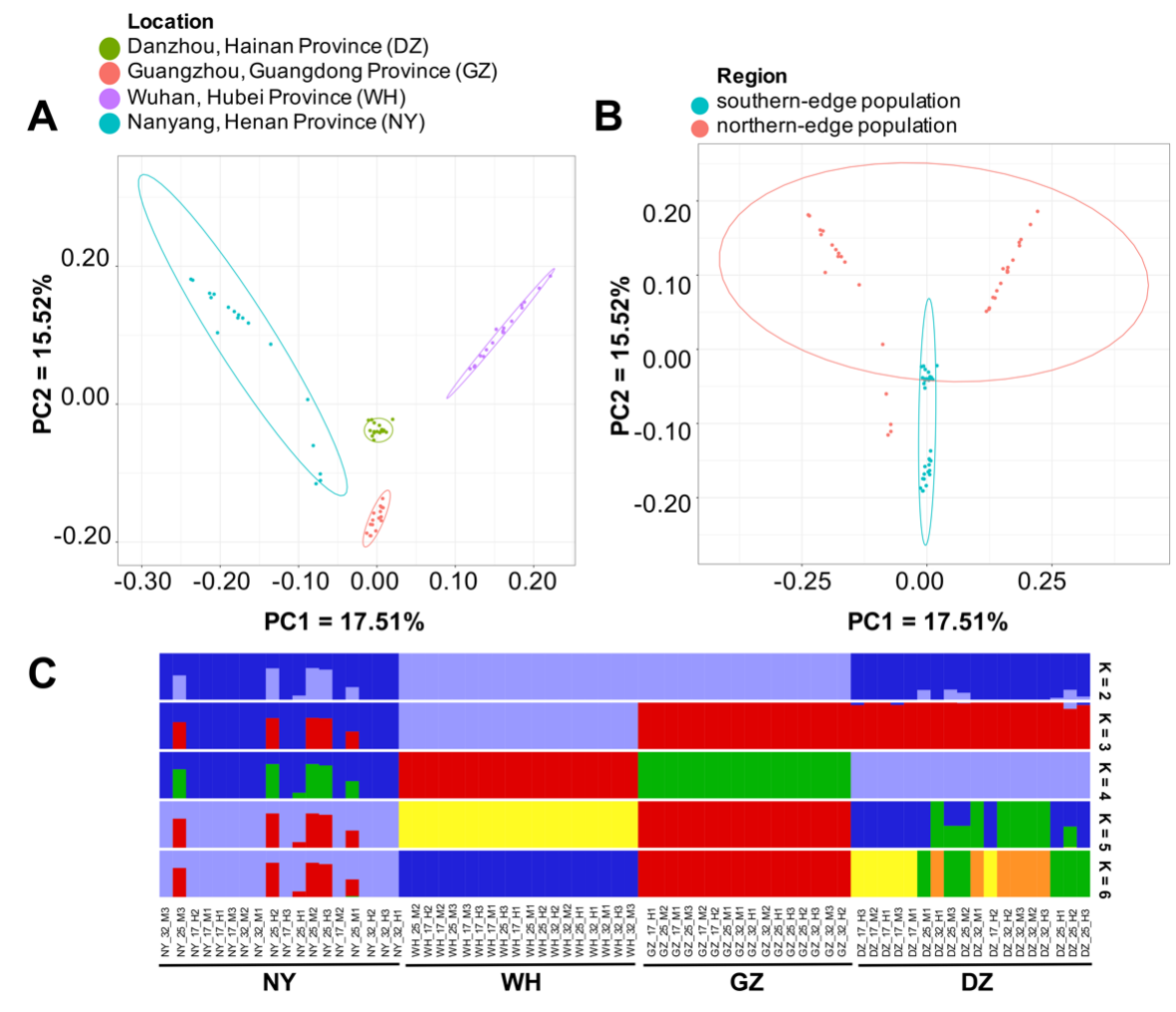
**

**Figure S9.** Population genetic structure of *Bactrocera dorsalis* between northern-edge and southern-edge populations. (**A**) Principal component analysis (PCA) plot showing individuals from four locations: Danzhou, Hainan Province (DZ), Guangzhou, Guangdong Province (GZ), Wuhan, Hubei Province (WH), and Nanyang, Henan Province (NY); (**B**) PCA plot of the same individuals grouped into their respective regional clusters: southern population (DZ and GZ) and northern population (WH and NY); (**C**) Population genetic structure based on ancestry fractions inferred by ADMIXTURE for when K = 2 to 6. Each vertical bar represents an individual, and the proportion of each color indicates the estimated ancestry contribution from K genetic clusters.

**
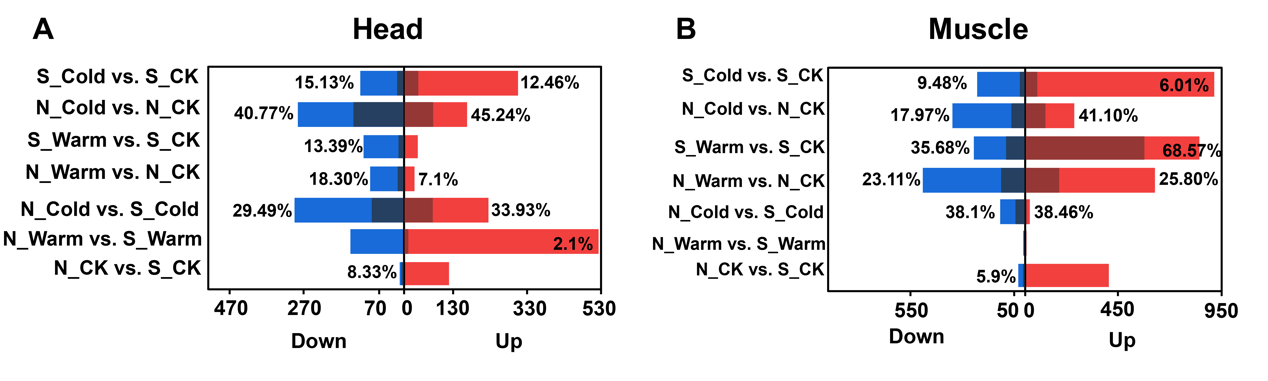
**

**Figure S10.** The number of DEGs and the proportion regulated by miRNA across seven pairwise differential expression comparisons in **(A)** head and **(B)** muscle tissues.

**
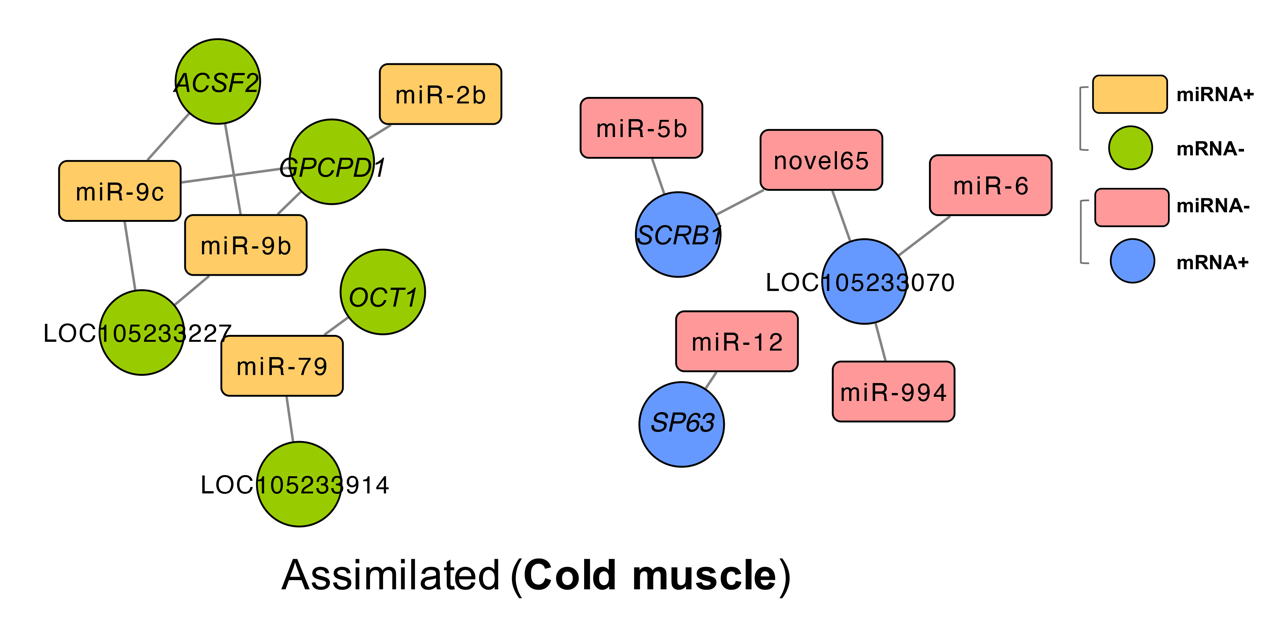
**

**Figure S11.** Predicted interaction network between miRNAs and genes involved in transcript expression plasticity evolution shown in **Figure 2A**.


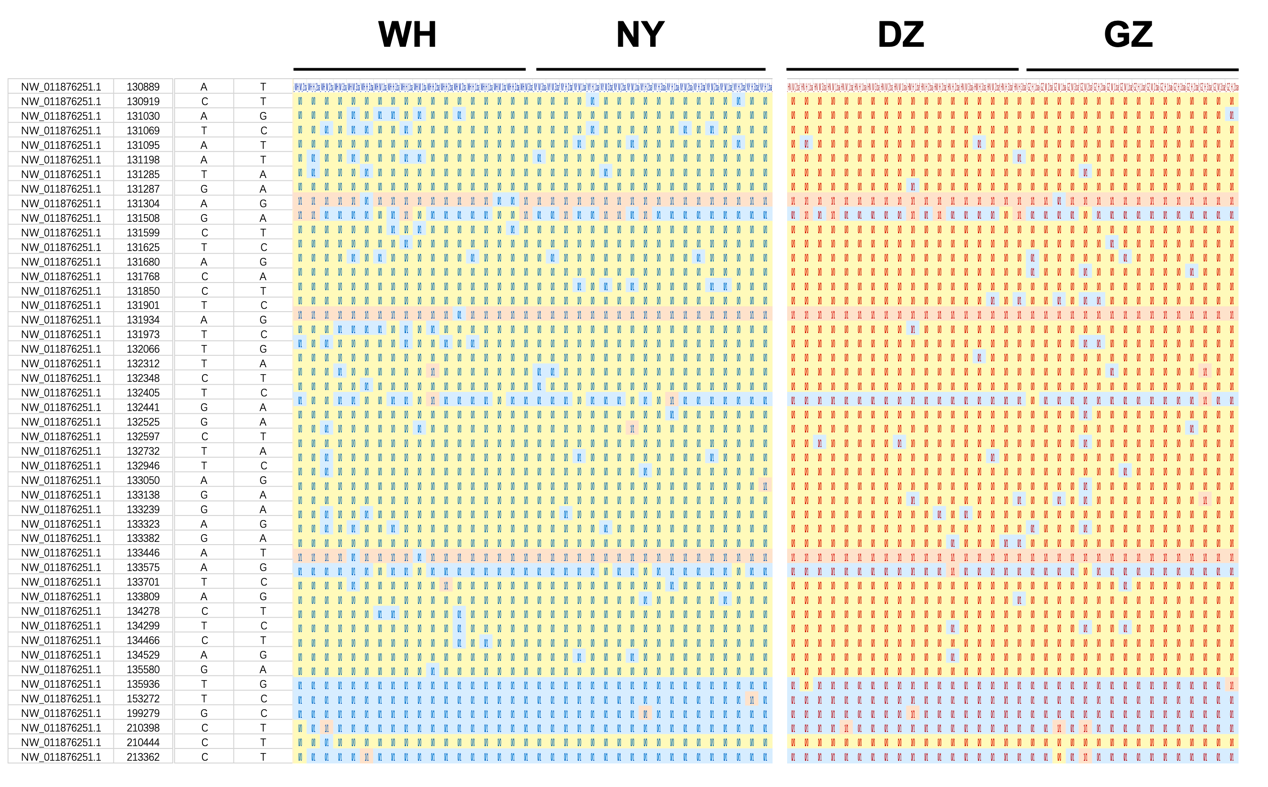


**Figure S12.** Single nucleotide polymorphism (SNP) analysis of the *thw* gene region based on RNA‑seq data from southern‑edge (DZ and GZ) and northern‑edge (WH and NY) populations of *Bactrocera dorsalis*. The heatmap displays allele frequencies across populations, with colors representing individual genotypes: homozygous for the reference allele (0/0, yellow), heterozygous (0/1, blue), and homozygous for the alternative allele (1/1, red).


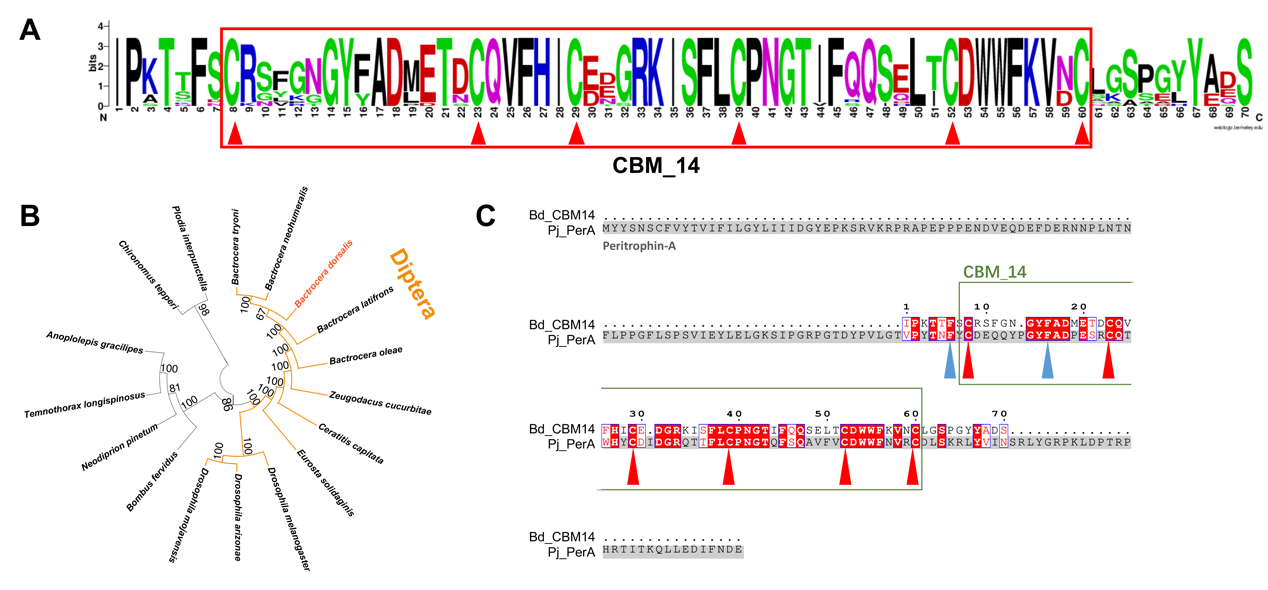


**Figure S13.**  Sequence conservation, phylogenetic analysis, and structural characterization of the *thw*gene in *Bactrocera dorsalis*. (**A**) Sequence logo of the CBM_14 domain in *B. dorsalis* THW aligned with homologous domains from 16 other insect species. Residue height reflects conservation level (bits). (**B**) Neighbor-joining phylogenetic tree of full-length THW protein sequences from *B. dorsalis* and 16 other insects. Bootstrap support values (>50%) are shown at nodes. Diptera species form a monophyletic clade (highlighted in orange). (**C**) Structural comparison between the CBM_14 domain of *B. dorsalis* THW and the canonical chitin-binding Peritrophin-A domain from *Popillia japonica*, with red triangles indicating six conserved cysteine residues critical for domain stability and blue triangles marking chitin-binding residues (F/Y) involved in substrate recognition, showing the CBM_14 domain as the chitin-binding module within the larger Peritrophin-A domain architecture.


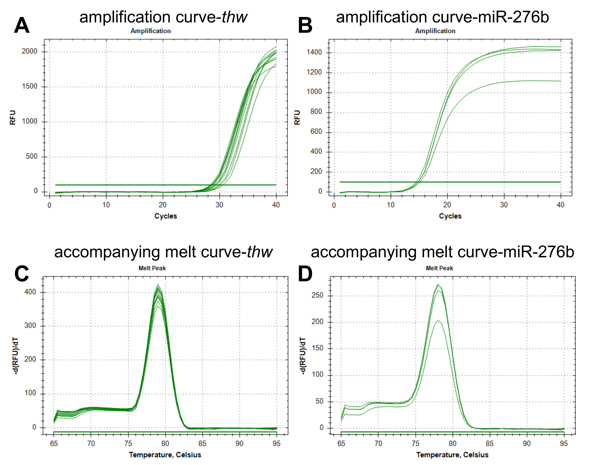


**Figure S14.** Melt curve and amplification plot for the primers used in quantitative real-time PCR (qRT-PCR).

**
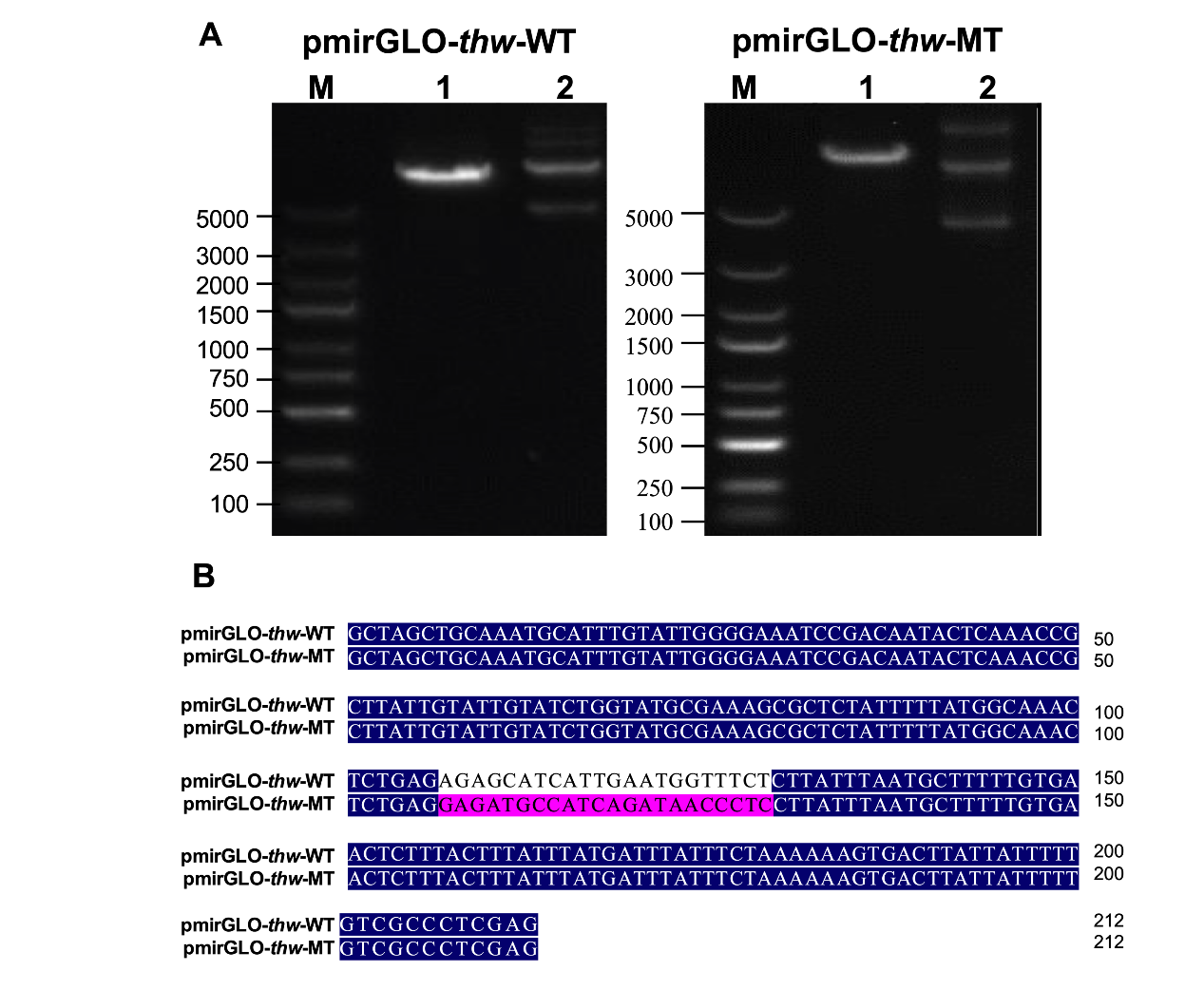
**

**Figure S15.** (**A**) Construction of the recombinant plasmid of pmirGLO-*thw*. Line M: DNA Marker (100~5000 bp); Line 1: Plasmid digested by XhoI; Line 2: Plasmid DNA. (**B**) Sequencing results for the recombination plasmid of pmirGLO-*thw*-WT, and pmirGLO-*thw*-MT in *B. dorsalis*.


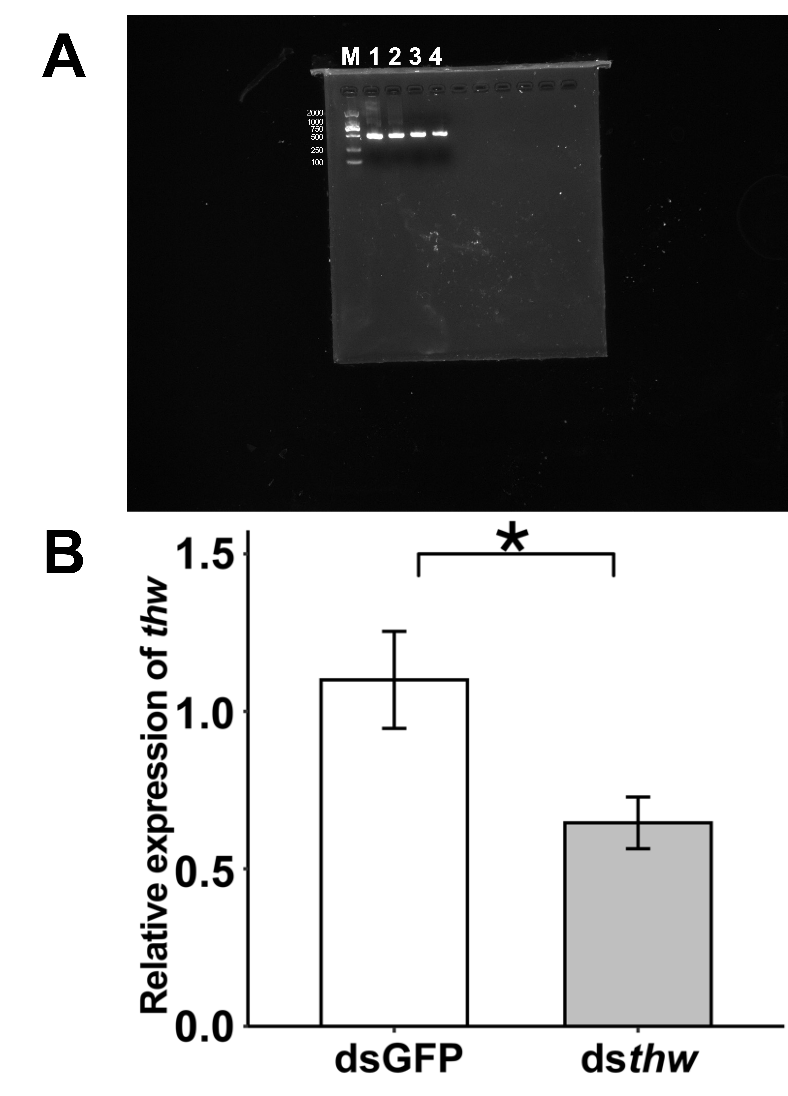


**Figure S16.** (**A**) Specific primers amplification results of *thw* in *B. dorsalis*. Line M: DNA Marker (100~2000 bp); Line 1-4: Four replicates. (**B**) Relative expression level of *thw* in *B. dorsalis* after RNA interference (n = 12, *p* = 0.017, Independent sample t-test). Data are presented as mean values and standard errors (mean ± SEM). Asterisks “*” above the bars represent significant differences at *p* < 0.05.

**Table S1.** The mean values and standard errors (mean ± SEM) for critical thermal limit (CT_min_ and CT_max_), adult body size (femur length and wing area), and body mass (adult weight) for each Region (southern-edge and northern-edge regions) and Thermal acclimation (17 ℃, 25 ℃ and 32 ℃). Significance of variation among the six acclimation groups for each trait was tested with one-way ANOVA (*p* < 0.05). Detailed statistical results are available in **Dataset S10**.

| **Trait** | **Region** | **Thermal acclimation** | **Mean ± SE** |
| --- | --- | --- | --- |
| CT_min_ (℃) | southern-edge | 17 ℃ | 4.13 ± 0.085 e |
|  |  | 25 ℃ | 9.73 ± 0.16 b |
|  |  | 32 ℃ | 10.81 ± 0.18 a |
|  | northern-edge | 17 ℃ | 4.40 ± 0.032 d |
|  |  | 25 ℃ | 7.59 ± 0.084 c |
|  |  | 32 ℃ | 9.65 ± 0.14 b |
| CT_max_ (℃) | southern-edge | 17 ℃ | 42.84 ± 0.15 b |
|  |  | 25 ℃ | 41.84 ± 0.14 c |
|  |  | 32 ℃ | 43.86 ± 0.15 a |
|  | northern-edge | 17 ℃ | 42.80 ± 0.12 b |
|  |  | 25 ℃ | 42.25 ± 0.067 c |
|  |  | 32 ℃ | 43.87 ± 0.056 a |
| femur length (mm) | southern-edge | 17 ℃ | 1.73 ± 0.010 d |
|  |  | 25 ℃ | 1.96 ± 0.012 a |
|  |  | 32 ℃ | 1.68 ± 0.010 e |
|  | northern-edge | 17 ℃ | 1.86 ± 0.011 b |
|  |  | 25 ℃ | 1.86 ± 0.011 b |
|  |  | 32 ℃ | 1.81 ± 0.012 c |
| wing area (cm^2^) | southern-edge | 17 ℃ | 0.090 ± 0.0011 de |
|  |  | 25 ℃ | 0.11 ± 0.0013 a |
|  |  | 32 ℃ | 0.087 ± 0.0014 e |
|  | northern-edge | 17 ℃ | 0.10 ± 0.0012 b |
|  |  | 25 ℃ | 0.098 ± 0.0012 c |
|  |  | 32 ℃ | 0.092 ± 0.0014 d |
| adult weight (mg) | southern-edge | 17 ℃ | 22.76 ± 0.47 d |
|  |  | 25 ℃ | 37.32 ± 0.49 a |
|  |  | 32 ℃ | 23.84 ± 0.49 d |
|  | northern-edge | 17 ℃ | 25.94 ± 0.51 c |
|  |  | 25 ℃ | 38.28 ± 0.54 a |
|  |  | 32 ℃ | 29.24 ± 0.50 b |

**Table S2**. Summary results from a factorial analysis of variance showing the effects of Region (southern-edge and northern-edge regions) and Thermal acclimation (17 ℃, 25 ℃ and 32 ℃) on critical thermal limit (CT_min_ and CT_max_), adult body size (femur length and wing area), and body mass (adult weight) using two-way ANOVA (*p* < 0.05).

| Trait | Effect | SS | d.f. | MS | *F* | *P* |
| --- | --- | --- | --- | --- | --- | --- |
| CT_min_ | Region | 112.53 | 1 | 112.53 | 95.57 | 1.67 × 10^-20^ |
|  | Thermal acclimation | 2668.53 | 2 | 1334.27 | 1133.21 | 1.68 × 10^-171^ |
|  | Region × Thermal acclimation | 107.55 | 2 | 53.78 | 45.67 | 1.06 × 10^-18^ |
| CT_max_ | Region | 1.80 | 1 | 1.80 | 1.74 | 0.19 |
|  | Thermal acclimation | 262.26 | 2 | 131.13 | 127.10 | 2.26 × 10^-44^ |
|  | Region × Thermal acclimation | 4.51 | 2 | 2.26 | 2.19 | 0.11 |
| femur length | Region | 0.34 | 1 | 0.34 | 26.34 | 3.82 × 10^-7^ |
|  | Thermal acclimation | 2.48 | 2 | 1.24 | 95.94 | 4.69 × 10^-37^ |
|  | Region × Thermal acclimation | 1.38 | 2 | 0.69 | 53.22 | 4.55 × 10^-22^ |
| wing area | Region | 7.31 × 10^-6^ | 1 | 7.31 × 10^-6^ | 0.078 | 0.78 |
|  | Thermal acclimation | 0.014 | 2 | 0.007 | 73.015 | 4.40 × 10^-27^ |
|  | Region × Thermal acclimation | 0.011 | 2 | 0.006 | 59.50 | 7.28 × 10^-23^ |
| adult weight | Region | 3855.85 | 1 | 3855.85 | 45.14 | 2.46 × 10^-11^ |
|  | Thermal acclimation | 60276.63 | 2 | 30138.31 | 352.80 | 6.47 × 10^-130^ |
|  | Region × Thermal acclimation | 1703.70 | 2 | 851.85 | 9.97 | 4.90 × 10^-5^ |

**Table S3.** General sequencing results for RNA-seq and miRNA-seq (Bd = *Bactrocera dorsalis*, 17 = cold acclimation, 25 = control temperature, 32 = warm acclimation, GZ = Guangzhou population, HN = Hainan population, NY = Nanyang population, WH = Wuhan population, H = head tissue, M = muscle tissue).

| **Type** | **Sample** | **Total Reads** | **Cutadapt reads (passing filters)** | **Cutadapt % (passing filters)** | **PCR duplicates (passing filters)** | **PCR duplicates % (passing filters)** |
| --- | --- | --- | --- | --- | --- | --- |
| mRNA | Bd17GZ_H1 | 28,213,033 | 28,208,963 | 99.99% | 11,705,290 | 41.49% |
| mRNA | Bd17GZ_H2 | 24,252,168 | 24,244,832 | 99.97% | 10,627,543 | 43.82% |
| mRNA | Bd17GZ_M1 | 27,782,495 | 27,775,197 | 99.97% | 8,255,112 | 29.71% |
| mRNA | Bd17GZ_M2 | 25,765,089 | 25,761,192 | 99.98% | 9,253,580 | 35.92% |
| mRNA | Bd17HN_H1 | 27,975,651 | 27,970,364 | 99.98% | 12,620,779 | 45.11% |
| mRNA | Bd17HN_H2 | 20,474,638 | 20,467,579 | 99.97% | 10,379,680 | 50.70% |
| mRNA | Bd17HN_H3 | 35,717,939 | 35,713,342 | 99.99% | 16,148,767 | 45.21% |
| mRNA | Bd17HN_M1 | 23,732,935 | 23,728,113 | 99.98% | 9,710,157 | 40.91% |
| mRNA | Bd17HN_M2 | 32,289,082 | 32,284,726 | 99.99% | 10,710,131 | 33.17% |
| mRNA | Bd17HN_M3 | 24,752,791 | 24,749,502 | 99.99% | 10,018,837 | 40.48% |
| mRNA | Bd17NY_H1 | 26,883,207 | 26,879,489 | 99.99% | 12,656,159 | 47.08% |
| mRNA | Bd17NY_H2 | 28,701,090 | 28,698,021 | 99.99% | 12,984,092 | 45.24% |
| mRNA | Bd17NY_H3 | 23,984,779 | 23,980,729 | 99.98% | 12,450,803 | 51.91% |
| mRNA | Bd17NY_M1 | 28,364,964 | 28,360,973 | 99.99% | 9,316,033 | 32.84% |
| mRNA | Bd17NY_M2 | 23,023,517 | 23,020,273 | 99.99% | 6,976,662 | 30.30% |
| mRNA | Bd17NY_M3 | 27,670,952 | 27,667,483 | 99.99% | 11,174,551 | 40.38% |
| mRNA | Bd17WH_H1 | 24,859,018 | 24,854,238 | 99.98% | 10,885,635 | 43.79% |
| mRNA | Bd17WH_H2 | 31,777,376 | 31,752,291 | 99.92% | 13,330,752 | 41.95% |
| mRNA | Bd17WH_H3 | 26,819,031 | 26,814,260 | 99.98% | 12,153,928 | 45.32% |
| mRNA | Bd17WH_M1 | 27,225,967 | 27,222,695 | 99.99% | 8,982,902 | 32.99% |
| mRNA | Bd17WH_M2 | 33,183,251 | 33,177,377 | 99.98% | 10,121,719 | 30.50% |
| mRNA | Bd17WH_M3 | 27,368,675 | 27,366,065 | 99.99% | 10,023,875 | 36.63% |
| mRNA | Bd25GZ_H1 | 20,528,078 | 20,524,241 | 99.98% | 9,672,147 | 47.12% |
| mRNA | Bd25GZ_H2 | 19,175,042 | 19,172,649 | 99.99% | 9,624,808 | 50.19% |
| mRNA | Bd25GZ_H3 | 21,609,023 | 21,600,709 | 99.96% | 9,946,752 | 46.03% |
| mRNA | Bd25GZ_M1 | 24,036,736 | 24,033,327 | 99.99% | 8,051,359 | 33.50% |
| mRNA | Bd25GZ_M2 | 28,338,043 | 28,334,144 | 99.99% | 7,922,940 | 27.96% |
| mRNA | Bd25GZ_M3 | 26,297,205 | 26,291,238 | 99.98% | 8,096,593 | 30.79% |
| mRNA | Bd25HN_H1 | 21,836,444 | 21,830,070 | 99.97% | 10,156,899 | 46.51% |
| mRNA | Bd25HN_H2 | 20,959,327 | 20,954,399 | 99.98% | 9,511,776 | 45.38% |
| mRNA | Bd25HN_H3 | 19,847,712 | 19,842,688 | 99.97% | 9,646,177 | 48.60% |
| mRNA | Bd25HN_M1 | 24,112,823 | 24,108,665 | 99.98% | 7,770,730 | 32.23% |
| mRNA | Bd25HN_M2 | 23,928,841 | 23,925,325 | 99.99% | 7,753,687 | 32.40% |
| mRNA | Bd25HN_M3 | 23,764,936 | 23,761,870 | 99.99% | 7,599,445 | 31.98% |
| mRNA | Bd25NY_H1 | 23,590,362 | 23,587,259 | 99.99% | 13,273,029 | 56.26% |
| mRNA | Bd25NY_H2 | 24,802,839 | 24,798,129 | 99.98% | 12,134,420 | 48.92% |
| mRNA | Bd25NY_H3 | 21,963,275 | 21,960,049 | 99.99% | 11,922,504 | 54.28% |
| mRNA | Bd25NY_M1 | 22,287,960 | 22,284,927 | 99.99% | 8,255,374 | 37.04% |
| mRNA | Bd25NY_M2 | 23,551,774 | 23,548,932 | 99.99% | 9,471,745 | 40.22% |
| mRNA | Bd25NY_M3 | 29,195,520 | 29,193,161 | 99.99% | 10,447,165 | 35.78% |
| mRNA | Bd25WH_H1 | 20,712,325 | 20,710,008 | 99.99% | 10,094,051 | 48.73% |
| mRNA | Bd25WH_H2 | 24,144,004 | 24,139,815 | 99.98% | 11,121,258 | 46.06% |
| mRNA | Bd25WH_H3 | 25,553,070 | 25,548,955 | 99.98% | 15,901,284 | 62.23% |
| mRNA | Bd25WH_M1 | 25,806,417 | 25,804,018 | 99.99% | 8,452,364 | 32.75% |
| mRNA | Bd25WH_M2 | 37,641,250 | 37,630,858 | 99.97% | 11,448,798 | 30.42% |
| mRNA | Bd25WH_M3 | 31,354,971 | 31,349,358 | 99.98% | 11,383,184 | 36.30% |
| mRNA | Bd32GZ_H1 | 22,291,908 | 22,283,711 | 99.96% | 10,089,299 | 45.26% |
| mRNA | Bd32GZ_H2 | 16,962,931 | 16,954,808 | 99.95% | 9,000,687 | 53.06% |
| mRNA | Bd32GZ_H3 | 20,008,484 | 20,003,104 | 99.97% | 9,934,451 | 49.65% |
| mRNA | Bd32GZ_M1 | 22,431,755 | 22,423,255 | 99.96% | 10,649,818 | 47.48% |
| mRNA | Bd32GZ_M2 | 22,531,769 | 22,525,228 | 99.97% | 10,264,213 | 45.55% |
| mRNA | Bd32GZ_M3 | 20,155,712 | 20,150,107 | 99.97% | 8,432,020 | 41.83% |
| mRNA | Bd32HN_H1 | 23,322,392 | 23,319,489 | 99.99% | 11,134,690 | 47.74% |
| mRNA | Bd32HN_H2 | 20,049,862 | 20,044,626 | 99.97% | 10,042,769 | 50.09% |
| mRNA | Bd32HN_H3 | 16,644,191 | 16,640,510 | 99.98% | 8,319,540 | 49.98% |
| mRNA | Bd32HN_M1 | 23,636,540 | 23,628,423 | 99.97% | 11,410,525 | 48.27% |
| mRNA | Bd32HN_M2 | 19,656,281 | 19,649,820 | 99.97% | 7,863,580 | 40.01% |
| mRNA | Bd32HN_M3 | 20,427,427 | 20,419,024 | 99.96% | 8,864,380 | 43.39% |
| mRNA | Bd32NY_H1 | 20,062,411 | 20,056,817 | 99.97% | 10,289,267 | 51.29% |
| mRNA | Bd32NY_H2 | 22,962,270 | 22,956,382 | 99.97% | 12,120,936 | 52.79% |
| mRNA | Bd32NY_H3 | 20,390,870 | 20,383,965 | 99.97% | 11,311,445 | 55.47% |
| mRNA | Bd32NY_M1 | 27,732,148 | 27,723,781 | 99.97% | 11,345,744 | 40.91% |
| mRNA | Bd32NY_M2 | 28,714,188 | 28,704,443 | 99.97% | 12,405,655 | 43.20% |
| mRNA | Bd32NY_M3 | 32,574,923 | 32,567,522 | 99.98% | 12,320,915 | 37.82% |
| mRNA | Bd32WH_H1 | 20,609,806 | 20,602,325 | 99.96% | 9,996,634 | 48.50% |
| mRNA | Bd32WH_H2 | 22,959,821 | 22,924,516 | 99.85% | 11,779,795 | 51.31% |
| mRNA | Bd32WH_H3 | 19,964,964 | 19,956,739 | 99.96% | 10,581,722 | 53.00% |
| mRNA | Bd32WH_M1 | 21,663,106 | 21,658,623 | 99.98% | 9,139,245 | 42.19% |
| mRNA | Bd32WH_M2 | 23,065,272 | 23,057,430 | 99.97% | 10,832,238 | 46.96% |
| mRNA | Bd32WH_M3 | 19,614,167 | 19,610,570 | 99.98% | 12,651,072 | 64.50% |

| **Type** | **Sample** | **Total Reads** | **Cutadapt reads(shorter than18 bp)** | **Cutadapt reads (longer than 24 bp)** | **Cutadapt reads (passing filters)** | **Cutadapt % (passing filters)** |
| --- | --- | --- | --- | --- | --- | --- |
| miRNA | Bd17GZ_H1 | 35,632,082 | 55,893 | 18,360,653 | 17,215,536 | 48.31% |
| miRNA | Bd17GZ_H2 | 35,199,766 | 162,724 | 12,262,700 | 22,774,342 | 64.70% |
| miRNA | Bd17GZ_M1 | 33,260,366 | 372,652 | 2,384,906 | 30,502,808 | 91.71% |
| miRNA | Bd17GZ_M2 | 34,852,415 | 462,464 | 2,940,246 | 31,449,705 | 90.24% |
| miRNA | Bd17HN_H1 | 34,733,748 | 121,067 | 13,597,340 | 21,015,341 | 60.50% |
| miRNA | Bd17HN_H2 | 35,155,607 | 153,739 | 7,006,024 | 27,995,844 | 79.63% |
| miRNA | Bd17HN_H3 | 36,205,992 | 138,666 | 10,771,052 | 25,296,274 | 69.87% |
| miRNA | Bd17HN_M1 | 34,026,294 | 340,065 | 2,940,476 | 30,745,753 | 90.36% |
| miRNA | Bd17HN_M2 | 33,617,630 | 404,561 | 1,576,316 | 31,636,753 | 94.11% |
| miRNA | Bd17HN_M3 | 30,145,280 | 344,028 | 733,877 | 29,067,375 | 96.42% |
| miRNA | Bd17NY_H1 | 34,944,309 | 137,096 | 6,597,222 | 28,209,991 | 80.73% |
| miRNA | Bd17NY_H2 | 35,924,074 | 115,282 | 7,509,978 | 28,298,814 | 78.77% |
| miRNA | Bd17NY_H3 | 33,321,266 | 70,731 | 9,924,009 | 23,326,526 | 70.00% |
| miRNA | Bd17NY_M1 | 29,904,415 | 323,458 | 1,565,190 | 28,015,767 | 93.68% |
| miRNA | Bd17NY_M2 | 34,433,909 | 328,379 | 2,684,566 | 31,420,964 | 91.25% |
| miRNA | Bd17NY_M3 | 32,519,845 | 313,957 | 3,610,581 | 28,595,307 | 87.93% |
| miRNA | Bd17WH_H1 | 33,099,462 | 47,671 | 12,719,849 | 20,331,942 | 61.43% |
| miRNA | Bd17WH_H2 | 33,448,406 | 204,936 | 2,937,024 | 30,306,446 | 90.61% |
| miRNA | Bd17WH_H3 | 35,959,496 | 107,014 | 10,546,763 | 25,305,719 | 70.37% |
| miRNA | Bd17WH_M1 | 29,990,068 | 270,384 | 628,249 | 29,091,435 | 97.00% |
| miRNA | Bd17WH_M2 | 29,191,689 | 316,175 | 615,680 | 28,259,834 | 96.81% |
| miRNA | Bd17WH_M3 | 28,767,819 | 316,536 | 1,764,860 | 26,686,423 | 92.76% |
| miRNA | Bd25GZ_H1 | 34,826,213 | 122,952 | 4,326,586 | 30,376,675 | 87.22% |
| miRNA | Bd25GZ_H2 | 32,328,505 | 117,157 | 2,713,634 | 29,497,714 | 91.24% |
| miRNA | Bd25GZ_H3 | 33,491,484 | 123,427 | 5,810,913 | 27,557,144 | 82.28% |
| miRNA | Bd25GZ_M1 | 24,569,696 | 313,002 | 1,246,491 | 23,010,203 | 93.65% |
| miRNA | Bd25GZ_M2 | 34,104,558 | 308,719 | 3,719,136 | 30,076,703 | 88.19% |
| miRNA | Bd25GZ_M3 | 31,191,099 | 316,023 | 1,360,568 | 29,514,508 | 94.62% |
| miRNA | Bd25HN_H1 | 34,587,257 | 78,940 | 11,819,632 | 22,688,685 | 65.60% |
| miRNA | Bd25HN_H2 | 33,613,343 | 148,835 | 3,631,880 | 29,832,628 | 88.75% |
| miRNA | Bd25HN_H3 | 34,035,797 | 64,165 | 12,307,444 | 21,664,188 | 63.65% |
| miRNA | Bd25HN_M1 | 31,515,395 | 263,431 | 3,099,059 | 28,152,905 | 89.33% |
| miRNA | Bd25HN_M2 | 32,507,984 | 292,696 | 4,996,809 | 27,218,479 | 83.73% |
| miRNA | Bd25HN_M3 | 30,720,566 | 255,526 | 762,528 | 29,702,512 | 96.69% |
| miRNA | Bd25NY_H1 | 35,954,335 | 80,526 | 10,742,877 | 25,130,932 | 69.90% |
| miRNA | Bd25NY_H2 | 35,192,810 | 129,792 | 1,667,162 | 33,395,856 | 94.89% |
| miRNA | Bd25NY_H3 | 34,594,407 | 79,997 | 7,406,545 | 27,107,865 | 78.36% |
| miRNA | Bd25NY_M1 | 30,378,990 | 227,950 | 1,174,368 | 28,976,672 | 95.38% |
| miRNA | Bd25NY_M2 | 33,376,182 | 148,617 | 1,581,233 | 31,646,332 | 94.82% |
| miRNA | Bd25NY_M3 | 32,595,685 | 132,937 | 1,972,659 | 30,490,089 | 93.54% |
| miRNA | Bd25WH_H1 | 33,774,587 | 66,449 | 9,470,093 | 24,238,045 | 71.76% |
| miRNA | Bd25WH_H2 | 33,350,310 | 181,946 | 287,626 | 32,880,738 | 98.59% |
| miRNA | Bd25WH_H3 | 33,091,517 | 143,320 | 4,318,321 | 28,629,876 | 86.52% |
| miRNA | Bd25WH_M1 | 35,353,104 | 242,128 | 2,968,338 | 32,142,638 | 90.92% |
| miRNA | Bd25WH_M2 | 30,842,812 | 245,026 | 1,518,946 | 29,078,840 | 94.28% |
| miRNA | Bd25WH_M3 | 32,784,428 | 334,283 | 1,248,070 | 31,202,075 | 95.17% |
| miRNA | Bd32GZ_H1 | 33,813,330 | 68,870 | 12,163,168 | 21,581,292 | 63.82% |
| miRNA | Bd32GZ_H2 | 33,062,793 | 186,126 | 2,433,630 | 30,443,037 | 92.08% |
| miRNA | Bd32GZ_H3 | 30,046,704 | 421,082 | 325,240 | 29,300,382 | 97.52% |
| miRNA | Bd32GZ_M1 | 31,730,735 | 458,523 | 2,569,968 | 28,702,244 | 90.46% |
| miRNA | Bd32GZ_M2 | 32,373,593 | 305,934 | 2,423,779 | 29,643,880 | 91.57% |
| miRNA | Bd32GZ_M3 | 32,991,171 | 428,494 | 4,866,303 | 27,696,374 | 83.95% |
| miRNA | Bd32HN_H1 | 35,057,826 | 238,411 | 3,596,962 | 31,222,453 | 89.06% |
| miRNA | Bd32HN_H2 | 34,308,223 | 132,444 | 5,675,258 | 28,500,521 | 83.07% |
| miRNA | Bd32HN_H3 | 34,839,781 | 146,847 | 7,310,963 | 27,381,971 | 78.59% |
| miRNA | Bd32HN_M1 | 33,325,463 | 344,106 | 451,909 | 32,529,448 | 97.61% |
| miRNA | Bd32HN_M2 | 35,566,846 | 282,909 | 7,843,981 | 27,439,956 | 77.15% |
| miRNA | Bd32HN_M3 | 33,723,127 | 217,843 | 5,958,593 | 27,546,691 | 81.68% |
| miRNA | Bd32NY_H1 | 34,134,960 | 153,803 | 2,360,105 | 31,621,052 | 92.64% |
| miRNA | Bd32NY_H2 | 33,278,739 | 164,149 | 7,802,303 | 25,312,287 | 76.06% |
| miRNA | Bd32NY_H3 | 32,943,263 | 60,184 | 13,558,042 | 19,325,037 | 58.66% |
| miRNA | Bd32NY_M1 | 29,856,950 | 524,014 | 1,031,571 | 28,301,365 | 94.79% |
| miRNA | Bd32NY_M2 | 33,669,452 | 221,132 | 7,269,378 | 26,178,942 | 77.75% |
| miRNA | Bd32NY_M3 | 34,622,768 | 290,132 | 5,454,776 | 28,877,860 | 83.41% |
| miRNA | Bd32WH_H1 | 31,430,999 | 222,445 | 1,703,565 | 29,504,989 | 93.87% |
| miRNA | Bd32WH_H2 | 34,747,228 | 242,498 | 1,696,464 | 32,808,266 | 94.42% |
| miRNA | Bd32WH_H3 | 30,990,108 | 282,250 | 1,785,533 | 28,922,325 | 93.33% |
| miRNA | Bd32WH_M1 | 35,666,043 | 178,246 | 18,076,866 | 17,410,931 | 48.82% |
| miRNA | Bd32WH_M2 | 34,841,261 | 199,668 | 18,118,665 | 16,522,928 | 47.42% |
| miRNA | Bd32WH_M3 | 35,804,417 | 339,503 | 15,791,967 | 19,672,947 | 54.95% |

**Table S4.** Mapping ratio results for RNA-seq and miRNA-seq (Bd = Bactrocera dorsalis, 17 = cold acclimation replicate, 25 = control temperature replicate, 32 = warm acclimation replicate, GZ = Guangzhou population, HN = Hainan population, NY = Nanyang population, WH = Wuhan population, H = head tissue, M = muscle tissue).

**RNA-seq:**

| **Sample** | Aligned concordantly 1 time | Overall alignment rate |
| --- | --- | --- |
| Bd17GZ_H_1 | 80.51% | 85.42% |
| Bd17GZ_H_2 | 79.66% | 84.87% |
| Bd17GZ_M_1 | 86.83% | 89.93% |
| Bd17GZ_M_2 | 85.23% | 89.20% |
| Bd17HN_H_1 | 77.99% | 83.34% |
| Bd17HN_H_2 | 75.84% | 81.74% |
| Bd17HN_H_3 | 80.21% | 84.88% |
| Bd17HN_M_1 | 82.50% | 86.87% |
| Bd17HN_M_2 | 83.86% | 88.06% |
| Bd17HN_M_3 | 84.07% | 88.50% |
| Bd17NY_H_1 | 78.05% | 84.00% |
| Bd17NY_H_2 | 80.43% | 85.66% |
| Bd17NY_H_3 | 78.48% | 83.99% |
| Bd17NY_M_1 | 85.42% | 89.74% |
| Bd17NY_M_2 | 87.36% | 90.82% |
| Bd17NY_M_3 | 82.27% | 87.00% |
| Bd17WH_H_1 | 79.18% | 84.61% |
| Bd17WH_H_2 | 81.10% | 86.06% |
| Bd17WH_H_3 | 80.01% | 85.45% |
| Bd17WH_M_1 | 85.79% | 89.70% |
| Bd17WH_M_2 | 86.01% | 90.12% |
| Bd17WH_M_3 | 85.37% | 89.31% |
| Bd25GZ_H_1 | 80.32% | 84.97% |
| Bd25GZ_H_2 | 80.46% | 85.13% |
| Bd25GZ_H_3 | 79.20% | 84.06% |
| Bd25GZ_M_1 | 86.19% | 89.94% |
| Bd25GZ_M_2 | 87.35% | 90.80% |
| Bd25GZ_M_3 | 85.82% | 89.34% |
| Bd25HN_H_1 | 78.65% | 83.92% |
| Bd25HN_H_2 | 79.57% | 84.59% |
| Bd25HN_H_3 | 79.59% | 84.76% |
| Bd25HN_M_1 | 85.84% | 89.94% |
| Bd25HN_M_2 | 85.71% | 89.43% |
| Bd25HN_M_3 | 84.88% | 89.08% |
| Bd25NY_H_1 | 79.36% | 84.67% |
| Bd25NY_H_2 | 80.01% | 84.91% |
| Bd25NY_H_3 | 77.93% | 82.75% |
| Bd25NY_M_1 | 84.14% | 88.07% |
| Bd25NY_M_2 | 84.91% | 88.86% |
| Bd25NY_M_3 | 84.44% | 88.80% |
| Bd25WH_H_1 | 77.39% | 82.80% |
| Bd25WH_H_2 | 79.47% | 84.69% |
| Bd25WH_H_3 | 71.80% | 75.95% |
| Bd25WH_M_1 | 84.92% | 88.94% |
| Bd25WH_M_2 | 84.80% | 88.92% |
| Bd25WH_M_3 | 82.45% | 86.13% |
| Bd32GZ_H_1 | 79.42% | 84.70% |
| Bd32GZ_H_2 | 77.29% | 82.19% |
| Bd32GZ_H_3 | 79.45% | 84.38% |
| Bd32GZ_M_1 | 82.06% | 85.48% |
| Bd32GZ_M_2 | 81.76% | 85.13% |
| Bd32GZ_M_3 | 83.96% | 87.80% |
| Bd32HN_H_1 | 77.98% | 83.85% |
| Bd32HN_H_2 | 79.59% | 84.58% |
| Bd32HN_H_3 | 80.66% | 85.67% |
| Bd32HN_M_1 | 80.54% | 84.60% |
| Bd32HN_M_2 | 85.29% | 88.75% |
| Bd32HN_M_3 | 84.55% | 88.40% |
| Bd32NY_H_1 | 80.01% | 85.19% |
| Bd32NY_H_2 | 79.58% | 84.53% |
| Bd32NY_H_3 | 79.45% | 84.61% |
| Bd32NY_M_1 | 83.87% | 87.62% |
| Bd32NY_M_2 | 82.66% | 86.82% |
| Bd32NY_M_3 | 83.73% | 87.34% |
| Bd32WH_H_1 | 79.50% | 84.91% |
| Bd32WH_H_2 | 79.68% | 84.99% |
| Bd32WH_H_3 | 79.18% | 84.39% |
| Bd32WH_M_1 | 84.52% | 88.53% |
| Bd32WH_M_2 | 83.18% | 87.31% |
| Bd32WH_M_3 | 73.43% | 77.27% |

**miRNA-seq:**

| ID | genome | hairpin | ncRNA | repeat | mRNA | intron |
| --- | --- | --- | --- | --- | --- | --- |
| Bd17GZ_H1 | 59.90% | 0.20% | 17.72% | 0.34% | 28.09% | 7.66% |
| Bd17GZ_H2 | 62.66% | 0.14% | 25.52% | 0.34% | 33.38% | 7.30% |
| Bd17GZ_M1 | 78.70% | 0.05% | 20.56% | 0.40% | 50.61% | 7.00% |
| Bd17GZ_M2 | 82.72% | 0.05% | 22.45% | 0.39% | 50.47% | 6.78% |
| Bd17HN_H1 | 54.23% | 0.25% | 29.28% | 0.29% | 13.81% | 4.99% |
| Bd17HN_H2 | 54.99% | 0.22% | 21.58% | 0.31% | 16.25% | 5.51% |
| Bd17HN_H3 | 49.66% | 0.23% | 20.97% | 0.29% | 12.96% | 4.93% |
| Bd17HN_M1 | 87.06% | 0.03% | 17.15% | 0.44% | 56.27% | 7.06% |
| Bd17HN_M2 | 82.47% | 0.04% | 22.32% | 0.44% | 52.99% | 6.70% |
| Bd17HN_M3 | 85.00% | 0.03% | 26.54% | 0.38% | 54.92% | 6.89% |
| Bd17NY_H1 | 46.64% | 0.22% | 24.67% | 0.31% | 12.69% | 4.51% |
| Bd17NY_H2 | 52.22% | 0.16% | 20.88% | 0.30% | 24.97% | 6.74% |
| Bd17NY_H3 | 48.42% | 0.20% | 19.04% | 0.30% | 20.91% | 6.37% |
| Bd17NY_M1 | 78.14% | 0.05% | 31.23% | 0.35% | 47.17% | 6.69% |
| Bd17NY_M2 | 73.69% | 0.06% | 24.99% | 0.34% | 43.18% | 6.80% |
| Bd17NY_M3 | 81.04% | 0.05% | 24.76% | 0.36% | 46.58% | 6.49% |
| Bd17WH_H1 | 52.01% | 0.20% | 14.28% | 0.33% | 26.07% | 7.32% |
| Bd17WH_H2 | 63.28% | 0.12% | 29.14% | 0.28% | 30.42% | 6.80% |
| Bd17WH_H3 | 58.09% | 0.16% | 20.67% | 0.32% | 28.87% | 6.78% |
| Bd17WH_M1 | 80.06% | 0.05% | 28.04% | 0.30% | 46.68% | 6.23% |
| Bd17WH_M2 | 81.27% | 0.05% | 29.97% | 0.31% | 45.04% | 6.06% |
| Bd17WH_M3 | 80.96% | 0.05% | 28.09% | 0.34% | 49.42% | 6.46% |
| Bd25GZ_H1 | 58.97% | 0.15% | 24.41% | 0.26% | 25.20% | 6.55% |
| Bd25GZ_H2 | 55.91% | 0.24% | 26.73% | 0.25% | 12.01% | 5.18% |
| Bd25GZ_H3 | 49.65% | 0.14% | 23.26% | 0.24% | 21.94% | 5.77% |
| Bd25GZ_M1 | 76.77% | 0.05% | 31.82% | 0.32% | 47.48% | 5.93% |
| Bd25GZ_M2 | 75.57% | 0.07% | 19.18% | 0.33% | 46.64% | 6.27% |
| Bd25GZ_M3 | 56.31% | 0.07% | 31.48% | 0.24% | 32.23% | 4.23% |
| Bd25HN_H1 | 49.49% | 0.24% | 20.16% | 0.30% | 16.02% | 6.31% |
| Bd25HN_H2 | 51.03% | 0.20% | 31.95% | 0.27% | 10.57% | 4.56% |
| Bd25HN_H3 | 49.68% | 0.23% | 18.07% | 0.30% | 17.17% | 6.43% |
| Bd25HN_M1 | 80.57% | 0.04% | 23.94% | 0.33% | 48.88% | 6.29% |
| Bd25HN_M2 | 73.94% | 0.06% | 22.13% | 0.37% | 45.47% | 6.11% |
| Bd25HN_M3 | 84.35% | 0.04% | 24.74% | 0.35% | 51.46% | 6.48% |
| Bd25NY_H1 | 50.92% | 0.19% | 20.49% | 0.26% | 21.00% | 6.41% |
| Bd25NY_H2 | 52.45% | 0.19% | 27.78% | 0.22% | 14.87% | 5.33% |
| Bd25NY_H3 | 46.19% | 0.24% | 21.82% | 0.28% | 10.94% | 4.73% |
| Bd25NY_M1 | 83.57% | 0.03% | 24.21% | 0.36% | 51.45% | 7.14% |
| Bd25NY_M2 | 50.22% | 0.19% | 29.52% | 0.22% | 14.17% | 4.83% |
| Bd25NY_M3 | 55.58% | 0.18% | 31.73% | 0.24% | 11.52% | 4.69% |
| Bd25WH_H1 | 45.76% | 0.27% | 18.82% | 0.30% | 9.96% | 4.64% |
| Bd25WH_H2 | 51.22% | 0.18% | 34.03% | 0.20% | 12.11% | 4.07% |
| Bd25WH_H3 | 56.98% | 0.15% | 26.58% | 0.25% | 23.03% | 6.34% |
| Bd25WH_M1 | 86.08% | 0.03% | 22.07% | 0.39% | 55.55% | 7.27% |
| Bd25WH_M2 | 82.22% | 0.03% | 26.51% | 0.34% | 53.10% | 7.32% |
| Bd25WH_M3 | 81.27% | 0.04% | 27.52% | 0.37% | 52.19% | 7.15% |
| Bd32GZ_H1 | 50.03% | 0.28% | 17.59% | 0.33% | 11.43% | 5.69% |
| Bd32GZ_H2 | 55.07% | 0.17% | 28.70% | 0.23% | 15.38% | 5.80% |
| Bd32GZ_H3 | 82.31% | 0.05% | 31.49% | 0.33% | 48.12% | 6.98% |
| Bd32GZ_M1 | 72.13% | 0.06% | 25.90% | 0.34% | 45.45% | 7.11% |
| Bd32GZ_M2 | 85.08% | 0.03% | 18.42% | 0.39% | 52.74% | 8.73% |
| Bd32GZ_M3 | 76.06% | 0.04% | 17.61% | 0.44% | 50.40% | 7.87% |
| Bd32HN_H1 | 59.27% | 0.20% | 25.50% | 0.30% | 14.66% | 5.77% |
| Bd32HN_H2 | 52.63% | 0.22% | 23.78% | 0.29% | 12.46% | 5.51% |
| Bd32HN_H3 | 51.72% | 0.21% | 22.76% | 0.28% | 11.82% | 5.32% |
| Bd32HN_M1 | 88.29% | 0.03% | 25.68% | 0.38% | 52.08% | 9.00% |
| Bd32HN_M2 | 83.50% | 0.03% | 12.58% | 0.41% | 52.27% | 9.11% |
| Bd32HN_M3 | 84.38% | 0.03% | 13.62% | 0.39% | 51.54% | 9.70% |
| Bd32NY_H1 | 56.27% | 0.15% | 28.36% | 0.27% | 22.00% | 6.86% |
| Bd32NY_H2 | 58.18% | 0.16% | 28.86% | 0.30% | 22.49% | 7.17% |
| Bd32NY_H3 | 44.84% | 0.30% | 20.40% | 0.34% | 11.21% | 5.28% |
| Bd32NY_M1 | 72.84% | 0.06% | 27.15% | 0.33% | 43.08% | 6.92% |
| Bd32NY_M2 | 74.88% | 0.05% | 13.87% | 0.43% | 49.79% | 8.20% |
| Bd32NY_M3 | 80.86% | 0.04% | 17.61% | 0.43% | 50.32% | 8.03% |
| Bd32WH_H1 | 57.82% | 0.18% | 29.61% | 0.25% | 12.87% | 4.83% |
| Bd32WH_H2 | 53.11% | 0.15% | 30.35% | 0.22% | 12.72% | 4.83% |
| Bd32WH_H3 | 57.39% | 0.14% | 33.11% | 0.25% | 15.67% | 5.06% |
| Bd32WH_M1 | 74.94% | 0.06% | 17.19% | 0.36% | 50.62% | 8.17% |
| Bd32WH_M2 | 80.71% | 0.04% | 15.60% | 0.40% | 52.23% | 8.93% |
| Bd32WH_M3 | 65.80% | 0.06% | 24.10% | 0.32% | 42.34% | 7.31% |

**Table S5.** Oligo nucleotides sequence of miR-276b in *Bactrocera dorsalis.*

| Name | Sequence (5’-3’) |
| --- | --- |
| miR-276b | UAGGAACUUCAUACCGUGCUCU |
| mimic-276b | AGAGCACGGUAUGAAGUGCUCU |
| mimic-NC | Sence: UCACAACCUCCUAGAAAGAGUAGA |
|  | Antisence: UCUACUCUUUCUAGGAGGUUGUGA |

**Table S6.** Primer details for the determination of expression levels of miR-276b and four target genes in *Bactrocera dorsalis.*

| Gene/miRNA name | Primer | Primer sequence (5’-3’) | Base number (bp) | Amplification size (bp) | Efficiency (%) | R^2^ |
| --- | --- | --- | --- | --- | --- | --- |
| *thw* | F | GAGACTGACTGCCAGGTATTTCAT | 24 | 133 | 98.8033 | 0.9684 |
|  | R | GTGAGCCGAGACAATTCACCT | 21 |  |  |  |
| miR-276b | F | TAGGAACTTCATACCGTGCTCT | 22 | 22 | 92.0423 | 0.9680 |
|  | R | GCAAATTCGTGAAGCGTTCCATA | 23 |  |  |  |
| *18s* | F | GCGAGAGGTGAAATTCTTGG | 20 | / | / | / |
|  | R | CGGGTAAGCGACTGAGAGAG | 20 |  |  |  |
| *U6* | F | AACGAGACGACGACAGAC | 18 | / | / | / |
|  | R | GCAAATTCGTGAAGCGTTCCATA | 23 |  |  |  |

**Table S7.** Primer details for template DNA amplification in dsRNA synthesis of *thw* in *Bactrocera dorsalis.*

| Gene name | Primer | Primer sequence (5’-3’) | Base number (bp) | Amplification size (bp) |
| --- | --- | --- | --- | --- |
| GFP | F | CACAAGTTCAGCGTGTCCG | 19 | 420 |
|  | R | GTTCACCTTGATGCCGTTC | 19 |  |
| *thw* | F | GAGCCACGCACCACAACCATT | 21 | 491 |
|  | R | TTCGCCAACTTGCCGTCCATATC | 23 |  |

**Supplementary Datasets**

**Dataset S1. Differentially expressed genes from seven pairwise comparisons in head and muscle.**

**Dataset S2. GO terms of genes associated with genes showing evolution of transcript expression plasticity.**

**Dataset S3. Genes within the yellow WGCNA module eigengenes for the head.**

**Dataset S4. GO terms of genes within WGCNA module eigengenes for the muscle.**

**Dataset S5. Hub genes related to thermal adaptation.**

**Dataset S6. Novel and known miRNAs identified in *Bactrocera dorsalis*.**

**Dataset S7. Differentially expressed miRNAs from seven pairwise comparisons in head and muscle.**

**Dataset S8. Regulation of differentially expressed miRNA on differential gene expression.**

**Dataset S9. Regulation of miRNA on the expression of frontloaded genes, assimilated genes and hub genes.**

**Dataset S10. Details statistic values in the figures.**

**Dataset S11. Bioinformatics scripts used for data processing and analysis in this study.**
