# Supplementary material for: Thermal Plasticity is Regulated by a Key MicroRNA During Range Expansion of an Invasive Fruit Fly
Source: Adv Sci (Weinh). 2026 Feb 24;13(23):e07662. doi: 10.1002/advs.202507662 (PMC13104144; doi:10.1002/advs.202507662)
Supplement: Supplementary file 2 — Supporting File: advs74381‐sup‐0002‐Data.zip. [file ADVS-13-e07662-s001.zip › advs74381-sup-0002-Data/advs202507662-sup-00012-Dataset S11.docx]

# ============================================

# **RNA-seq Alignment Pipeline**

# ============================================

# 1. Build genome index

hisat2-build -f ./GCF_000789215.1_ASM78921v2_genomic.fna ./genome

# 2. Align reads (example: sample 17-GZ-H-1)

hisat2 --new-summary -p 10 -k 1 --rna-strandness RF \

-x ./genome \

-1 ./cleandata/17-GZ-H-1_FRRB210061225-1a_1_val_1.fq.gz \

-2 ./cleandata/17-GZ-H-1_FRRB210061225-1a_2_val_2.fq.gz \

-S 17_GZ_H_1.sam

# 3. Convert SAM to sorted BAM

samtools sort -o 17_GZ_H_1.bam 17_GZ_H_1.sam

# 4. Index BAM file

samtools index 17_GZ_H_1.bam

# Optional: Remove intermediate SAM file

# rm 17_GZ_H_1.sam

**# SAMtools processing pipeline (duplicate marking + QC)**

samtools sort -n -o 17_GZ_H_1_namesort.bam 17_GZ_H_1.bam

samtools fixmate 17_GZ_H_1_namesort.bam 17_GZ_H_1_fixmate.bam

samtools sort -o 17_GZ_H_1_positionsort.bam 17_GZ_H_1_fixmate.bam

samtools markdup -S 17_GZ_H_1_positionsort.bam 17_GZ_H_1_markdup.bam

samtools flagstat 17_GZ_H_1_markdup.bam > 17_GZ_H_1_markdup.metrics.txt

**# Gene-level read counting with HTSeq**

htseq-count -f bam -r name -s reverse -a 10 -t exon -i gene_id -m union \

17_GZ_H_1_markdup.bam \

./genome.gtf \

> 17_GZ_H_1_markdup_htseq.txt

```

# ==================== **RNA-seq Differential Expression Analysis** ====================

# Analysis for Head tissue samples with factorial design (Area × Temperature)

# 1. Load required packages

library(tidyverse)

library(DESeq2)

# 2. Set working directory

setwd("/path/to/your/analysis_directory/H/")

# 3. Import data

gene_exp_H <- read.csv("countdata_all70_filter_H.csv", row.names = 1)

coldata_H <- read.delim("coldata_H.txt", row.names = 1)

# 4. Build DESeq2 object with factorial design

dds <- DESeqDataSetFromMatrix(

countData = gene_exp_H,

colData = coldata_H,

design = ~ Area + Tem + Area:Tem

)

# Set factor levels (reference groups)

dds$Area <- factor(dds$Area, levels = c("S", "N")) # South as reference

dds$Tem <- factor(dds$Tem, levels = c("25", "17", "32")) # 25°C as reference

# Run DESeq2 analysis

dds <- DESeq(dds)

# 5. Extract results for seven key comparisons

# 5.1 Interaction effects

res_AreaN_Tem17 <- results(dds, name = "AreaN.Tem17")

res_AreaN_Tem32 <- results(dds, name = "AreaN.Tem32")

# 5.2 Main temperature effects (South population)

res_low_Tem <- results(dds, contrast = c("Tem", "17", "25"))

res_high_Tem <- results(dds, contrast = c("Tem", "32", "25"))

# 5.3 Temperature effects in North population (main + interaction)

res_low_Tem_N <- results(dds, contrast = list(c("Tem_17_vs_25", "AreaN.Tem17")))

res_high_Tem_N <- results(dds, contrast = list(c("Tem_32_vs_25", "AreaN.Tem32")))

# 5.4 Regional differences under different conditions

res_NvsS_without_tre <- results(dds, contrast = c("Area", "N", "S"))

res_NvsS_with_low <- results(dds, list(c("Area_N_vs_S", "AreaN.Tem17")))

res_NvsS_with_high <- results(dds, list(c("Area_N_vs_S", "AreaN.Tem32")))

# 6. Filter and export significant DEGs (padj < 0.05, |log2FC| > 1)

results_list <- list(

AreaN_Tem17 = res_AreaN_Tem17,

AreaN_Tem32 = res_AreaN_Tem32,

low_Tem = res_low_Tem,

high_Tem = res_high_Tem,

low_Tem_N = res_low_Tem_N,

high_Tem_N = res_high_Tem_N,

NvsS_without = res_NvsS_without_tre,

NvsS_low = res_NvsS_with_low,

NvsS_high = res_NvsS_with_high

)

# Apply filtering and export

for (name in names(results_list)) {

# Filter significant genes

sig_genes <- subset(results_list[[name]], padj < 0.05 & abs(log2FoldChange) > 1)

# Export results

write.csv(results_list[[name]], file = paste0("DEG_H_", name, "_raw.csv"))

write.csv(sig_genes, file = paste0("DEG_H_", name, ".csv"))

# Print summary

cat("\n=== ", name, " ===\n")

print(dim(sig_genes))

}

# 7. Session info (for reproducibility)

sessionInfo()

# ==================== **miRNA-seq Processing Pipeline** ====================

# 1. Quality trimming and adapter removal

trim_galore --small_rna --length 18 --max_length 24 \

--stringency 3 --phred33 --cores 10 \

--dont_gzip -o ./cleandata/Bd17GZ_H1 Bd17GZ_H1A.fq

# 2. Process reads with mapper.pl

mapper.pl ./cleandata/Bd17GZ_H1/Bd17GZ_H1A_trimmed.fq \

-e -g 1G1 -h -m -s ./Bd17GZ_H1.fa

# 3. Map reads to reference genome

bowtie -f -n 1 -l 8 -a -m 5 --best --strata \

--al Bd17GZ_H1.mapped.fa \

./genome ./Bd17GZ_H1.fa \

> Bd17GZ_H1.genome.bwt 2> Bd17GZ_H1.genome.log

# 4. Annotate sRNA reads

perl srna_anno.pl -fa Bd17GZ_H1.mapped.fa \

-rfam ./Rfam/family.txt \

-mirna Bd17GZ_H1.miRNA.bwt \

-ncrna Bd17GZ_H1.ncRNA.bwt \

-intron Bd17GZ_H1.intron.bwt \

-repeat Bd17GZ_H1.repeat.bwt \

-exon Bd17GZ_H1.exon.bwt \

-outpre Bd17GZ_H1.out

# 5. miRNA discovery with miRDeep2

# 5.1 Prepare input files

mapper.pl all_2off.reads.fasta -c -o 10 -r 5 \

-p ./genome -t reads_collapsed_vs_genome.arf

# 5.2 Run miRDeep2

miRDeep2.pl all_2off.reads.fasta ./genome.fa \

reads_collapsed_vs_genome.arf \

./mature.bdo.fa ./mature.Hexapoda.fa ./hairpin.bdo.fa \

2> report.log

# 6. Combine known and novel miRNA sequences

cat ./hairpin.bdo.fa ./novel_out.precursor.fa > all.hairpin.fa

cat ./mature.bdo.fa ./novel_out.mature.fa > all.mature.fa

# 7. Merge all sample reads (simplified)

cat Bd*.mapped.fa > all.reads.fa

# 8. Quantify miRNA expression

quantifier.pl -p all.hairpin.fa -m all.mature.fa \

-r all.reads.fa -g 0

# 9. Format expression matrices

# Read counts

awk 'NR==1 || !($1 in seen){print; seen[$1]=1}' \

miRNAs_expressed_all_samples_*.csv | \

cut -f1,5- > miRNAs_expressed.count.txt

# TPM values

awk 'NR==1 || !($1 in seen){print; seen[$1]=1}' \

miRNAs_expressed_all_samples_*.csv | \

sed 's/(norm)//g' | \

cut -f1,$((5+(NF-4)/2))- > miRNAs_expressed.TPM.txt
